# Bimetallic Single-Atom Catalysts for Water Splitting

**DOI:** 10.1007/s40820-024-01505-2

**Published:** 2024-09-25

**Authors:** Megha A. Deshmukh, Aristides Bakandritsos, Radek Zbořil

**Affiliations:** 1 Nanotechnology Centre, Centre for Energy and Environmental Technologies, VŠB–Technical University of Ostrava, 17. listopadu 2172/15, 708 00 Ostrava-Poruba, Czech Republic; 2 Regional Centre of Advanced Technologies and Materials, Czech Advanced Technology and Research Institute (CATRIN), Palacký University Olomouc, Šlechtitelů 241/27, 783 71 Olomouc – Holice, Czech Republic

**Keywords:** Single-atom catalysts, Single-atom dimers, Hydrogen evolution, Oxygen evolution, Water splitting

## Abstract

Green hydrogen from water splitting has emerged as a critical energy vector with the potential to spearhead the global transition to a fossil fuel-independent society. The field of catalysis has been revolutionized by single-atom catalysts (SACs), which exhibit unique and intricate interactions between atomically dispersed metal atoms and their supports. Recently, bimetallic SACs (bimSACs) have garnered significant attention for leveraging the synergistic functions of two metal ions coordinated on appropriately designed supports. BimSACs offer an avenue for rich metal–metal and metal–support cooperativity, potentially addressing current limitations of SACs in effectively furnishing transformations which involve synchronous proton–electron exchanges, substrate activation with reversible redox cycles, simultaneous multi-electron transfer, regulation of spin states, tuning of electronic properties, and cyclic transition states with low activation energies. This review aims to encapsulate the growing advancements in bimSACs, with an emphasis on their pivotal role in hydrogen generation via water splitting. We subsequently delve into advanced experimental methodologies for the elaborate characterization of SACs, elucidate their electronic properties, and discuss their local coordination environment. Overall, we present comprehensive discussion on the deployment of bimSACs in both hydrogen evolution reaction and oxygen evolution reaction, the two half-reactions of the water electrolysis process.

## Highlights

- Bimetallic single-atom catalysts (bimSACs) have garnered significant attention for leveraging the synergistic functions of the two metal active centers.
- This review focuses on the advancements in the field of bimSACs and their pivotal role in hydrogen generation via water splitting.
- State-of-the-art computational and physicochemical techniques for the analysis of bimSACs and their application in electrocatalytic water splitting are discussed.

## Graphical abstract

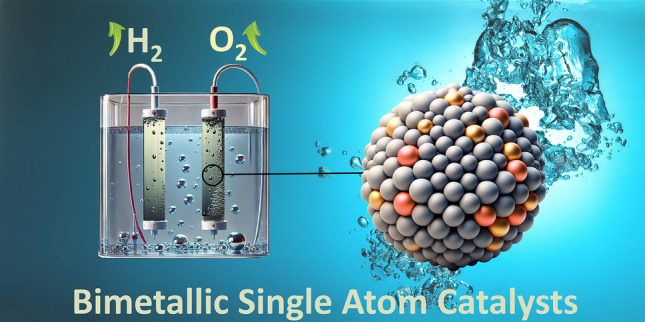

## Introduction

**Fig. 1:**
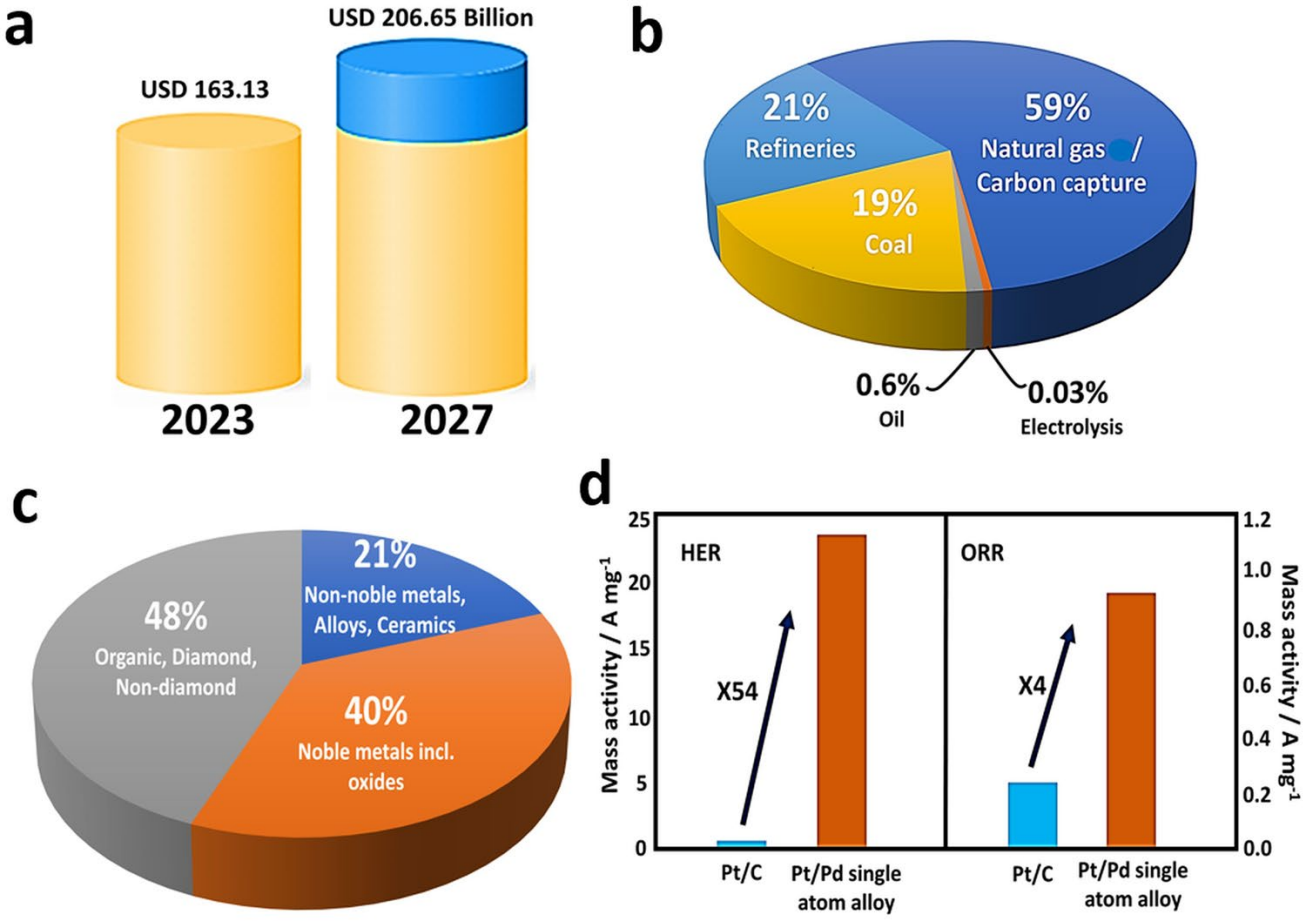
**a** Global hydrogen market projection in terms of value. **b** Comparison of hydrogen production sources. **c** Materials as electrocatalysts for water splitting. **d** Catalytic activity of conventional Pt nanocatalysts and Pt_1_/Pd SAAs. Reproduced with permission from Ref. [20] Copyright 2019 American Chemical Society

Hydrogen plays a pivotal role in our energy security and toward a sustainable and technologically advanced society [1]; thus, hydrogen-related technologies are gaining unparalleled momentum globally [2, 3]. For over 200 years, hydrogen has been intertwined with energy, from fueling the earliest internal combustion engines to its pivotal role in today's refining industry [4, 5]. Hydrogen emits no direct pollutants or greenhouse gases [6], is lightweight, storable, and is a high-energy density carrier in liquid state [7, 8]. Globally, the demand for green hydrogen as a renewable energy source is surging [9], augmented by increasing government investments and subsidies advocating for clean fuels [10–13]. Hydrogen can be considered as an eco-friendly alternative to fossil fuels [14], which is likely to drive the global market in the years to come [8, 10–12]. According to recent analyses, the value of the global green hydrogen market is projected to expand from USD (United States Dollar) 163.13 billion to USD 206.65 billion in the foreseeable future [15–17] (Fig. 1a) [10, 12, 13, 18, 19].

On this basis, the next generation of hydrogen production technologies demands the use of sustainable, and earth-abundant catalysts [21–23], as well as renewable electricity resources for electrocatalytic processes [10, 12]. Renewable hydrogen can be produced from fossil fuels, biomass, water, and other resources. Notably, natural gas is currently the predominant source for hydrogen production (Fig. 1b). Water splitting technologies that split water into hydrogen and oxygen [24] provide a simple, yet efficient, greener, and promising method for hydrogen production [25], which could replace fossil-based hydrogen production (e.g., natural gas). However, overall water splitting (OWS) requires highly active and cost-effective electrocatalysts [26], with long-term stability for both hydrogen evolution reaction (HER) and oxygen evolution reaction (OER) [27]. Despite the tremendous efforts to find more efficient electrocatalysts for hydrogen production, precious metal-based nanoparticulate catalysts remain the most efficient ones [28, 29] (Fig. 1c). However, the high and continuously rising prices, and the limited natural reserves of precious metals hamper their large-scale application in electrocatalytic water splitting (EWS) [29]. Among the most essential catalyst features is maximizing the number of exposed active sites, which can be achieved by reducing the size of the active species (i.e., the nanoparticles (NPs)) and by improving their dispersion [19].

In this context, single-atom catalysts (SACs) with atomically distributed metal centers promise ultimate atom economy and efficiency [30], thus attracting worldwide scientific attention in the fields of photo-, electro-, and thermal catalysis, with almost 100% atom exposure [31], tunable electronic properties and coordination environment, recyclability, and improved performance compared to their NP counterparts [30]. SACs have brought prominent advancements in several electrochemical reactions including OER and HER [30]. For instance, ultrafine Pt NPs supported on N,S-co-doped porous carbon nanofibers (Pt–N,S-pCNFs) hybrids showed a HER overpotential (*η*) of 168 mV at 10 mA cm^−2^ [32]. On the other hand, Pt_1_/MoO_3−*x*_ SACs exhibited much higher activity compared to NPs. The *η* required to reach a current density (*j*) of 10 mA cm^−2^ for Pt_1_/MoO_3−*x*_/C was much lower (23.3 mV) [33]. Su et. al. developed a Pt-based SAC with high electrochemical activity for OER with *j* of 120 mA cm^−2^ at a low *η* of 405 mV [34]. In another example, iridium (Ir) SAC exhibited a high water oxidation activity with a low *η* of ~ 170 mV at 10 mA cm^−2^ current density [20]. Moreover, Zhang et al. reported Pt/Pd-based single-atom alloy (SAA) catalysts for electrochemical catalytic reactions, such as HER and oxygen reduction reaction (ORR), with improved activity compared to commercial Pt/C catalysts (Fig. 1d) [20]. Although these outstanding features of SACs already distinguish them within the realm of catalysts [35], there are several challenges that need to be addressed, which still hold them back from their widespread application [35, 36]. One of the major restrictions, pertinent to HER and OER, is an inadequate number of adsorption sites for reactions that require co-adsorption of multiple reactants, primarily because only one isolated atom is accessible in SACs [35, 36].

An efficient strategy to address such challenges is the introduction of additional single atoms (SAs) with different electronic properties (e.g., of element type, oxidation state or another coordination environment) in close proximity, to form hetero-bimetallic single-atom catalysts (hetero-bimSACs). This strategy can lead to strong synergistic interactions and functions between the asymmetrically deployed SA sites, such as polarized charge distribution with tunable electron accumulation or deficiency around the metal centers [20], or preference for coordination with different reactants [37]. The discrete atomic microenvironment (i.e., the local coordination environment) controls the electronic state of the catalytic centers. This dynamic provides a potent mechanism to fine-tune the performance of the SAs toward high activity, selectivity, and stability, especially in bimSACs for EWS technologies [30]. Moreover, the possibility of engineering unsaturated coordination environments at the active sites can further improve the catalytic performance in water splitting. This is achieved by creating conditions that are both sterically and energetically optimal for the sorption and desorption of reactants [38]. The solid supports where the SAs are embedded also render SACs reusable and thus sustainable, green, and cost-effective, unlike the case of homogeneous molecular catalysts. Finally, the solid supports offer an extended and multilevel coordination sphere, not attainable in homogeneous molecular catalysts, opening the door for unique charge transfer phenomena and otherwise unattainable electronic or valence states of the SAs [39, 40].

**Fig. 2:**
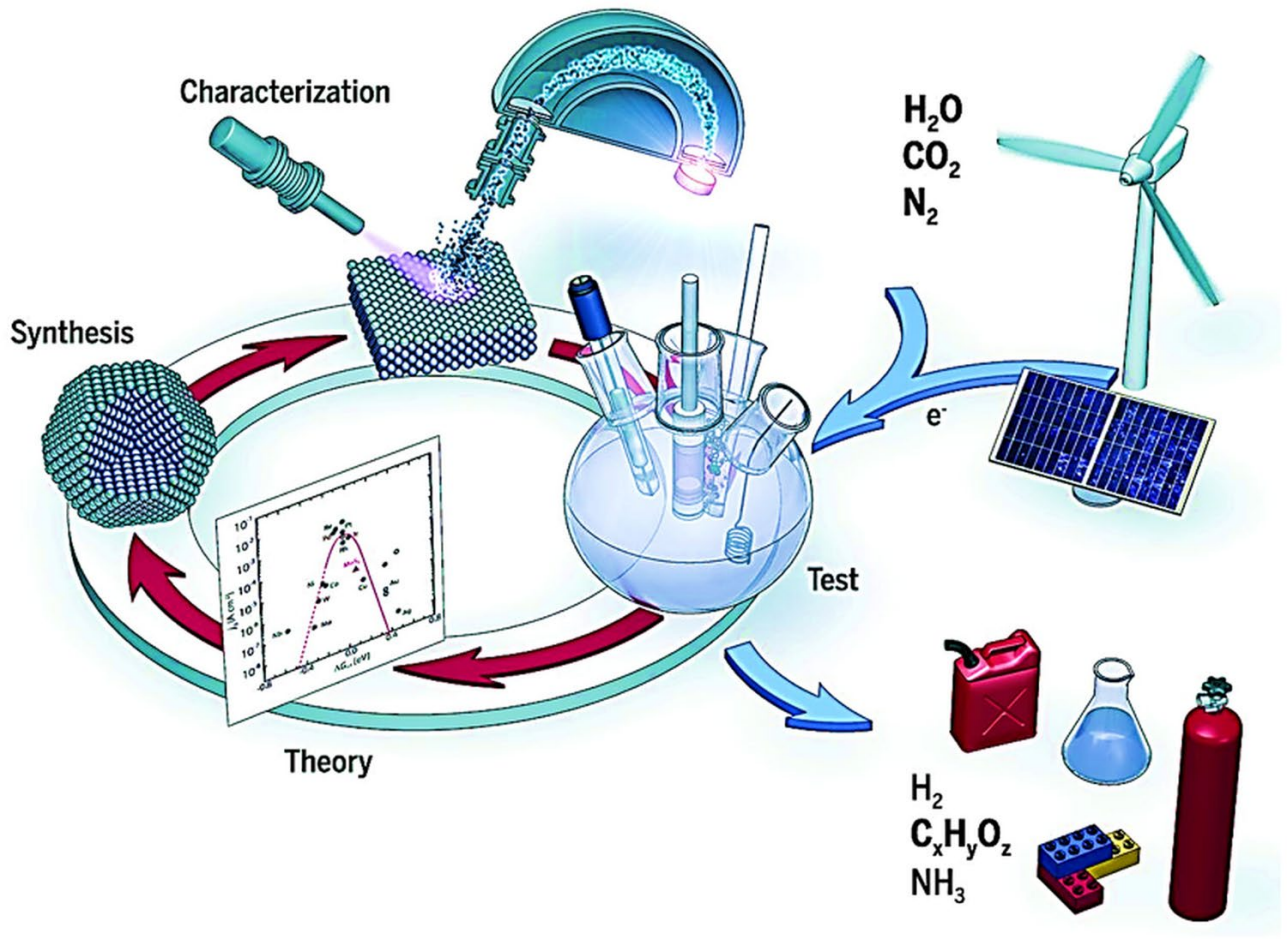
Electrochemical water splitting, reduction of CO_2_ and N into high- and added-value products using advanced catalysts and renewable electricity sources. The concerted theoretical and experimental analyses can guide the design and development of high-performance electrocatalysts that are pivotal for advancing such technologies. Reproduced with permission from Ref. [41]. Copyright 2024 AAS

Systematic and concerted theoretical and experimental efforts can illuminate the overarching trends and principles guiding the development of advanced catalysts [41]. These insights play a crucial role in advancing our understanding of catalysts’ functionality, paving the way for designing sophisticated catalysts. Such advanced catalysts may exhibit intricate synergistic functions that mirror some of the inherent multilevel synergisms observed in the naturally evolved enzymatic biocatalysts, but with improved stability in harsh reaction environments. Such insights are pivotal to effectively furnish complex, multiredox, and multicomponent electrochemical transformations with high selectivity, essential for clean energy technologies, not only for water splitting, but also for the production of hydrogen peroxide (H_2_O_2_), the reduction of carbon dioxide (CO_2_) and nitrogen (N), in addition to HER and OER (Fig. 2) [41]. In this review paper, we delve into in-depth analysis of bimSACs in electrocatalysis, particularly for the HER, OER, and OWS. This work highlights that the synergistic interactions and distinct active sites of bimSACs can overcome the limitations of traditional SACs materials and conventional bimetallic systems. The review provides a thorough examination of how these dual-atom configurations enhance catalytic efficiency, stability, and tunability. Furthermore, it offers new insights into their electronic structures and local coordination environments. Additionally, the paper explores advanced theoretical characterization techniques, including density functional theory (DFT), ab initio molecular dynamics (AIMD), and machine learning, providing a detailed understanding of bimSACs' behavior and performance.

## Types of SACs

Heterogeneous catalysts utilizing SAs have been present in various forms for a long time, particularly when considering enzymes [42]. Synthetic catalysts have also been developed, albeit scarcely, but date back at least as early as 1979, when Yates et al. reported on the “catalytic decomposition of formaldehyde on single rhodium atoms” which were supported on alumina. Although advanced characterization techniques, such as aberration-corrected electron microscopy, were not available, infrared studies relying on the vibrations of carbonyls’ (like carbon monoxide) interactions with the metal were powerful enough for a first understanding [43]. In 1995 in Nature, T. Maschmeyer et al. working on surface organometallic chemistry, a field in part resembling SACs, reported the grafting of an organometallic Ti complex on silica [44]. Although the heterogenization of preformed complexes has been a well-known topic, it is particularly noteworthy that this pre-catalyst was thermally treated to remove the organic ligand, leading to a highly active single-metal atom. Importantly, X-ray absorption spectroscopy (XAS) was utilized to delineate the coordination environment of the active site, understanding its important role in the activity. SACs have nowadays gained enormous attention within the research communities as well as in the industrial research and development sectors owing to their remarkable intrinsic properties [45].

The atomically precise metal distribution in SACs, their exceptional catalytic activities, and product selectivities render these materials as a unique bridge between homogeneous and heterogeneous catalysis [46, 47], offering promising advancements in the field [45]. The recent examples of SACs-based materials are stimulating as they are being successfully applied in a wide variety of catalytic reactions, while offering deeper insights into the working mechanisms due to simpler structure of the active centers in comparison with nanoparticulate heterogeneous systems [48–50]. The electronic structure of SACs predominantly depends on the architecture of their coordination environment and the properties of specific host [51, 52]. Strong binding to the host via ionic or, mainly, covalent interactions leading to SA anchoring plays a significant role in promoting substantial charge transfer effects, for example [52]. The adsorption strength of SAs depends on their cationic or anionic nature, as well as on the frontier orbital energies and the level of interactions of the host’s coordination sites with the frontier orbitals of the metal atoms, leading to enhancement or, in other cases, to hindering the catalytic performance [52].

The concept and realm of SACs is not just limited to the case of having atoms of one type of metal with 100% dispersion [52–54]. Isolated atoms from different metal elements create intriguing SAC systems, while understanding the effects of nuclearity such as in SAC dimers is crucial [54, 55]. Adding or removing an atom in the vicinity of another one can significantly alter the properties in a non-scalable manner due to the quantum confinement effects of electrons in metals and their interactions with the host material [52, 54]. However, achieving control and stable dimer SAC species of precise nuclearity, as well as distinguishing between them presents significant challenges [52]. The strategic assembly of two isolated metal atoms of different elemental origin leads to hetero-bimSACs, furnishing dual active sites that promote reactions involving the co-adsorption of multiple reactants [56]. This approach overcomes the limitations of single isolated atoms [57, 58]. Recent studies suggest that tuning the coordination sites of SACs to include sulfur (S) or phosphorus (P), or by introducing neighboring atoms to create bimSACs, can significantly modulate the electronic structure of these catalysts and enhance their intrinsic activity. This improvement is attributed to the unique atomic interface and the synergistic effects between the dual-metal sites, allowing for cooperative and concerted functions between different reactants [59–62].

Alloying is an effective strategy to fine-tune the geometric, ligand, and strain effects of metal catalysts [63–65]. This approach also helps to bring into practice the bifunctional mechanisms that modulate the electronic structure of these catalysts [63, 64]. However, metal alloys often exhibit scaling effects because they contain continuous sites corresponding to the individual constituent elements [66]. As a result, it is challenging to decrease the activation barrier of intermediates and weaken the binding energy of key intermediates in bulk alloys [67]. Nanoparticulate surfaces may also decrease selectivity owing to different activities of the exposed crystal planes and diverse interfaces with the supports. However, bimSACs have the potential to break linear scaling relationships in electrocatalysis. This is due to their unique ability to decouple dissociation (e.g., water splitting) and reaction sites (e.g., bond formation between two oxygen or two hydrogen atoms), offering a distinct advantage over traditional catalysts [65]. BimSACs demonstrate the ability to maintain a free, atom-like electronic structure, even when embedded on the surface of the host materials [68]. This characteristic distinguishes them from traditional alloys [65, 68]. Another key difference related to the single atomic structure in bimSACs is that metal–metal and metal–support interactions become more pronounced and more sensitive to the local coordination environment. In nanoalloys, perturbations on the orbital energies and density of states (DOS) become smeared out by those of the nanoassembly as material in its whole, where the electronic states are delocalized over the entire metal lattice, leading to averaged electronic properties of the two metals. The charge distribution is more homogeneous, as the electrons are free to move across the metallic lattice, unlike the case of bimSACs [28]. BimSACs often exhibit lower DOS values near the Fermi level compared to their bulk forms [69]. The significant variation in electronic structure between bimSACs and their bulk counterparts related to the reduction in the valence band also results from the mixing of valence bands between the SAs and the host materials [69]. This narrowing effect is particularly pronounced for 3*d* bimSACs [30]. The combination of two metal atoms can alter the d-band center of both SAs influencing the adsorption strength of reactants and intermediates [30], ultimately affecting the resulting catalytic activities [69]. BimSACs are capable of breaking the constraints of the Brønsted–Evans–Polanyi (BEP) relationship, which describes a linear dependence between the activation barrier and the reaction energy for a chemical conversion resulting in improved catalytic performance [70].

**Fig. 3:**
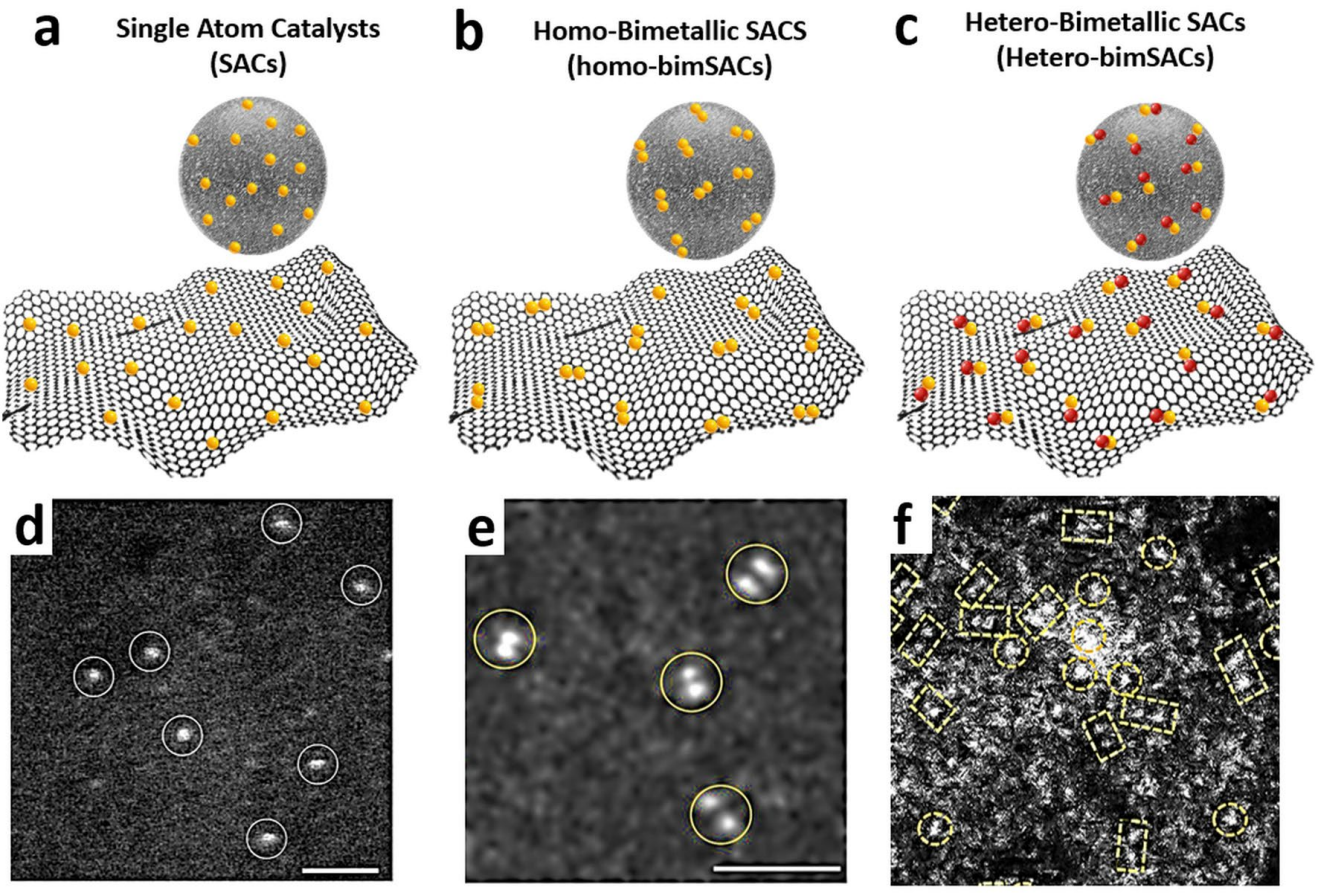
**a-c** Schematic of SACs, homo-bimSACs, and hetero-bimSACs. Aberration-corrected high-angle annular dark-field scanning transmission electron microscopy (HAADF-STEM) images of **d** SAC Pt_1_/graphene. **e** Homo-Pt_2_/graphene. Reproduced with permission from Ref. [74]. Copyright 2017 Springer Nature. **f** Aberration-corrected HAADF-STEM images of hetero-NiCo SACs. Reproduced with permission from Ref. [61]. Copyright 2017 Springer Nature

Broadly bimSACs can be categorized into two types, homo-bim SACs and hetero-bimSACs. The homo-bimSACs possess symmetric dimeric active sites and hetero-bimSACs asymmetric. In hetero-bimSACs, each SA in the dimeric unit is of different elemental origin or different electronic state (e.g., of different oxidation state) [56]. Thus, unlike SACs (Fig. 3a, d), homo-bimSACs (Fig. 3b, e) have well distributed single-metal atom dimers of the same chemical nature, while hetero-bimSACs (Fig. 3c, f) possess a pair of two different adjacent metal atoms, often bonded with each other [56, 71, 72], with interdependent and or correlated electronic structures of two metal sites. These features, as previously described, enhance the catalytic properties by altering the binding energy and electronic interactions in general with the reactants, products, and key reaction intermediates [36, 73].

### SACs, Homo-BimSACs, and Hetero-BimSACs

In bimSACs, the metal–metal bonds can be classified as nonpolar and polar bonds, considering homo- and heteronuclear metal sites [75], and are proficient at offering abundant reactive adsorption sites exhibiting diverse local charge distribution [56]. The metal–metal bond and the electronic structure of bimetallic centers boost catalytic performance by altering the binding energy of reactants [75], affording flexible adsorption configurations for reaction intermediates, and produce inter-site synergistic effects due to metal–metal and metal–support interactions [75]. The isolated adjacent active sites not only offer dual adsorption sites but also synergistically enhance catalytic activity and selectivity to a considerable extent. This can be achieved due to the enriched variety of geometric structures that can be statistically explored by the involved reaction species, significantly boosting the structural flexibility of adsorbed species and concerted intermediate formation, as well as by tuning the electronic properties and eventually the electrocatalytic properties in bimSACs [75]. Homo-SACs with identical metal atoms (Fig. 3b, e) and symmetric charge distribution are less capable of offering structural deformation and asymmetric charge density, unlike the case of hetero-bimSACs, which link different single-metal atoms [75]. Thus, hetero-bimSACs (Fig. 3c, f) may significantly promote perturbation in the electronic configuration owing to the metal–metal bond and correlated electronic states [76, 77], inducing charge polarization and extension of the electronic states near the Fermi level [76]. The electronic perturbation and the correlation between the metal SAs also lead to prominent charge density gradients in these sites generating high local torque and effective activation of linear molecules, more particularly, due to improved atomic orbitals overlapping [56]. In certain complex reactions concerning multistep proton–electron transfer, the homo- and hetero-bimSACs offer the possibility for the adsorption of reaction intermediates with different configurations, thus being able to facilitate the chemical transformations via multiple catalytic mechanisms [75]. Hetero-bimSACs take advantage of two different active sites to catalyze complicated chemical processes having multiple reaction steps and intermediate states [56]. Acidic ORR activity of dual-metal-nitrogen-carbon (DM–N–C) catalysts was studied for two M_1_M_2_N_6_ and one M_1_M_2_N_8_ models (M = Mn, Fe, Co, Ni, Cu, and Zn) with 63 active centers [78]. The thermal stability of the catalysts was estimated by calculating the binding energies, and the results implied that most of the dual-metal sites are stable within the N-doped graphene layers [78]. It was also revealed that the M_1_M_2_ antibonding center could facilitate the O–O bond cleavage and meanwhile mitigate the *OH desorption issue, resulting to very high catalytic activity [78]. In another example, the hydroxyl group modified dual-metal active site ((HO)_2_–M_1_M_2_ /DG, where M_1_ and M_2_ are Ni, Co, or Fe) on N-doped graphene networks revealed remarkable catalytic activity for both OER and ORR, better than that of isolated metal atoms [42]. Moreover, the distinct atoms in the asymmetric sites not only activate linear molecules but can also potentially break the traditional linear scaling relationships to overcome the kinetic energy barriers during the catalytic reactions [56]. In this regard, PtRu hetero-bimSACs supported on N-doped graphene revealed the lowest hydrogen adsorption free energy of − 0.07 eV [79]. Furthermore, it was discovered that the scaling relationship based on the dissociative chemisorption energy of water scales linearly with kinetic barriers, serving as a unique activity descriptor for bimSACs [79]. The disruption of the traditional linear scaling at the transition state on an asymmetric dual active site provides ample design flexibility [56, 80], thus particularly enhancing their unique ability to activate small molecules like N_2_, CO, CO_2_, and H_2_O, which is highly desirable [56]. Benefiting from the structural advantages of dual-metal atom compositions, various types of asymmetric centers have already been explored across numerous catalytic schemes [81, 82]. For example, in the indirect, coordination-induced catalytic interactions in Ru-Pt bimSAC, it was observed that, regardless of intermetallic coordination deficiency in the first and second shell of Ru-Pt bimSACs and charge distribution effects, the catalyst showed enhanced hydrogen formation rate compared to their individual counterparts [83].

## Synthesis Strategy of BimSACs

The controlled synthesis of bimSACs is particularly challenging due to two major obstacles. The first obstacle is achieving uniform distribution of single-metal atoms on the support surface without causing aggregation [84]. The second challenge is carefully controlling the bonding and coordination environment for each individual atom [84]. The precise synthesis of bimSACs is a notably complex task. The choice of both precursors and substrates plays critical role [85]. Synthesis of hetero-bimSACs is notably more intricate than homo-bimSACs [85, 86]. The effects of confinement and molecule anchoring considerably influence the stability and rational configuration of bimSACs during synthesis [87, 88]. Moreover, optimal exposure and availability of active sites are also critical factors which influence the catalytic performance of bimSACs [84]. “Top-down” and “bottom-up” methods are the two main synthetic approaches. The most frequently explored methods for bimSACs synthesis are the high-temperature pyrolysis and atomic layer deposition (ALD). Impregnation–adsorption method is a highly promising approach for the efficient production of bimSACs [89].

### Pyrolysis Method

To produce atomically dispersed catalysts such as SACs, bimSACs pyrolysis is known as one of the most effective and efficient techniques. Coordination compounds, such as metal–organic frameworks (MOFs), are extensively studied as precursors for the pyrolysis process [37, 84, 85]. This methodology influences the structure of coordination compounds, where metal-containing nodes are uniformly distributed by organic ligands [37, 84, 85]. This uniform distribution can inhibit the aggregation of metals during pyrolysis. Additionally, the robust porous structure of MOFs can confine infused precursors within their pores [37, 84, 85]. The host–guest approach is a widely adopted technique for inserting metal cations into MOFs or other porous structures, such as carbons, graphene and its derivatives [90–92]. This method facilitates the creation of metal sites in the final product [84, 92]. Liang et al. employed an efficient approach for the controlled synthesis of Ni_2_ bimSACs by transforming the ligands of the precursor [93]. During the synthesis, it was observed that a higher concentration of the dinuclear complex precursor and elevated pyrolysis temperatures led to the formation of Ni atom clusters [93]. This clustering reduced the catalytic activity and selectivity of the catalyst [93]. Recently, Niu et al. explored the synthesis of a library of bimSACs, including both homo-bimSACs and hetero-bimSACs, by encapsulating a macrocyclic metal complex (M_1_M_2_L) into the cavity of ZIF-8 using the confinement pyrolysis method [94]. In this approach, the Robson-type macrocyclic ligands provided a multifunctional coordination platform compatible with embedding various combinations of hetero- and homo-bimSACs. The limited internal size of the porous carbon framework, which acts as an encapsulation shell, imposes spatial constraints on the macrocyclic complex. This effectively prevents adverse thermal migration and agglomeration during subsequent high-temperature treatments, thereby preserving the structure of the bimSACs to a large extent [95]. A Fe–Mo–N–C bimSAC bifunctional electrocatalyst was developed using a one-step, template-free pyrolysis method, which eliminates the need for auxiliary metals and post-synthetic treatments [96]. The synthesized catalyst exhibited enhanced electrocatalytic performance for the OER/ORR. This improvement was attributed to the modification of the electronic structure around the metal atoms, induced by the presence of neighboring metal atoms [96]. Fe–Ni bimSACs were uniformly distributed on nitrogen-doped porous carbon (N–C) polyhedrals (FeNi-DSAs-PNCH) using a microwave-assisted adsorption followed by a template-free and ligand-free pyrolysis process. This method resulted in enhanced ORR activity and improved long-term stability [97]. In another example, Liu et al., constructed Cu–Zn bimetallic SA (Cu/PMCS) by a two-step hydrothermal pyrolysis method using Cu nitrate trihydrate (Cu(NO_3_)_2_·3H_2_O), Zn hexahydrate (Zn(NO_3_)_2_·6H_2_O) as precursors [98]. To produce high-quality Fe_2_ dimers on N–C, Ye et al. encapsulated binuclear Fe_2_(CO)_9_ into the pores of ZIF-8 before pyrolysis [99]. The harsh conditions of pyrolysis typically pose significant challenges for maintaining structural control, and the frequent coexistence of SAs can further complicate the process [85].

The presence of these SAs has remarkable impact on the performance of bimSACs, as demonstrated in electrocatalysis studies [85]. Theoretically, Co–Zn bimSACs should suppress the two-electron pathway (2e^−^) from O_2_ to H_2_O_2_ during the ORR [85, 100]. However, a significant amount of H_2_O_2_ was produced over the Co-Zn bimSACs with the presence of SAs [100]. This indicates that more precise control over the pyrolysis process is necessary to achieve the desired outcomes [85]. During the synthesis of bimSACs, the coordination environment of the supports is crucial. However, existing methods struggle to precisely tailor the coordination geometry due to the pyrolysis process, which is typically performed at temperatures above 800 °C [37, 85, 86, 88]. As a result, the only adjustable parameters are the types and amounts of heteroatoms in the supports [85]. In addition to MOFs, N is also employed as the most conventional heteroatom to modify C supports during synthesis of SACs and bimSACs [101, 102]. Despite extensive research into the synthesis of bimSACs using pyrolysis methods, there is still room for further improvement.

### Atomic Layer Deposition

Atomic layer deposition (ALD) is widely explored technique for thin films and nanomaterials synthesis due to its sequential, and its operation principle which exclusively involves surface chemistry [103]. ALD allows for precise control over the atomic layers of the materials being formed. Consequently, this technique is also gaining attention for the preparation of SACs and bimSACs [103]. The precise control over the formation of catalysts with SAs makes ALD a powerful tool for achieving atomically precise ultrafine metal clusters, including bimetallic sites [103]. This precision allows for in-depth investigation of the relationship between atomic structure and catalytic performance [74]. ALD relies on two sequential, self-limiting surface reactions at the molecular level, separated by inert gas purging [104–106]. The exclusive characteristic permits ALD to construct catalytic materials from the bottom up, regularly, and specifically, on high-surface-area substrates [104–106]. In the ALD process for creating bimSACs, two cycles are essential [85]. This allows the second metal to be selectively deposited onto the previously deposited metal, enabling precise control over the bimetallic sites [85]. Pt-Ru dual-metal dimers prepared on nitrogen-doped carbon nanotubes (NCNTs) are a typical example [103]. During the ALD process, the Pt precursor, (methylcyclopentadienyl)-platinum (IV) (MeCpPtMe_3_), initially absorbed and reacted mainly with the N atoms on NCNTs. This reaction forms a strong metal-support interaction through chemical bonding [103]. HAADF-STEM confirmed the presence of isolated Pt atoms. For Ru deposition, the ALD temperature was 270 °C, which was higher than Pt ALD [103]. Therefore, Pt SAs in ALD chamber were maintained at 270 °C for 1 h to investigate the stability of the Pt SAs, which was indeed confirmed. Then, the Pt–Ru dimers were formed by ALD of Ru on Pt SAs [103]. Yan et al. fabricated Pt_2_ bimSACs using a bottom-up approach on a graphene support. This was achieved by depositing Pt atoms sequentially on phenol-related O_2_ anchor sites through Pt ALD [74]. The presence of Pt_2_ dimers in the samples was confirmed by both HAADF-STEM and X-ray absorption fine structure (XAFS) analyses [74]. There are generally limited reports on the use of ALD for the preparation of bimSACs. However, the existing studies pave the way for the rational design of bimSACs with significant catalytic activity and stability through such surface-selective synthetic technique.

### Impregnation–Adsorption

The impregnation–adsorption method has also been used to prepare bimSACs. In this process, preselected binuclear metal complexes adsorb onto the surface of the supports through physical or chemical adsorption [107, 108]. For example, to form stable bimSACs and mitigate the thermal migration effects of atoms, Leng et al. reported an interfacial cladding engineering method [109]. In this approach, a cetyltrimethylammonium bromide (CTAB)-functionalized ZIF-8 was used as the support for metal atoms. The cyclopentadienyliron dicarbonyl dimer (Fe_2_ dimer) was sequentially immobilized onto the surface of the CTAB-functionalized ZIF-8 using an impregnation-adsorption procedure. After immobilizing the Fe_2_ dimer, dopamine was added and polymerized on the ZIF-8 surface to form a coating layer that encapsulates the Fe dimer [109]. In another example, Fe_2_/mp graphitic carbon nitride (g-C_3_N_4_) (Fe_2_/mp g-C_3_N_4_) bimSACs were prepared using the impregnation-adsorption approach. The preselected metal precursor, bis(dicarbonylcyclopentadienyliron) (Fe_2_O_4_C1_4_H_10_), was used in combination with mesoporous g-C_3_N_4_ as the support. This catalyst was employed for the selective epoxidation of trans-stilbene to trans-stilbene oxide [110]. Barrio et al. prepared C_2_N-Fe bimSACs using wet impregnation method in methanol flux for electrocatalysis application [111]. C_2_N-like materials were used as supports, and Fe coordination was performed using FeCl_2_ through a reflux process, followed by a metalation reaction. Cao et al. developed a facile room temperature impregnation method to fabricate atomically dispersed dual-site Ru supported on S-doped carbon black [112]. Through mechanistic studies, it was revealed that the reported catalyst can synergistically boost water molecule capture, water dissociation, and hydrogen release [112]. A Pd_1_-Ru_1_ bimSAC was developed using the wetness impregnation method, with porous ionic polymers (PIPs) serving as supports and Ru acting as an “assistant” or co-catalyst to the Pd_1_ sites [113]. The strong ionic bond between the anionic single-metal sites and the cationic polymer framework, along with the synergistic interaction between the neighboring single sites Pd_1_ and Ru_1_, enabled the Pd_1_-Ru_1_/PIPs catalyst to exhibit excellent catalytic performance for acetylene dialkoxycarbonylation [113]. Ni and Fe bimSACs experience significant performance degradation under high current densities and high *η* [114]. To address this issue, Sun et al. [114] used ionic liquids with different cations or anions to regulate the micro-surface of NiFe–N–C through an impregnation method. Recently, Chen et al. [115] prepared Co_4_S_3_/Co_9_S_8_ nanosheets (Co_4_S_3_/Co_9_S_8_ NS) using a solvothermal approach combined with ultrasonic exfoliation. Subsequently, different amounts of Fe^3+^ ions were doped into nanosheets through a simple one-step impregnation method to form Fe-doped Co_4_S_3_/Co_9_S_8_ NS. The Cr^3+^ ions were then further doped to form bimetallic Fe/Cr co-doped Co_4_S_3_/Co_9_S_8_. Notably, the Fe^3+^ doping significantly enhanced the OER performance of Fe/Cr co-doped Co_4_S_3_/Co_9_S_8_ catalyst with improved electrocatalytic activity and stability [115]. These findings can provide solid and convincing examples toward the development of a broad variety and miscellaneous configurations for the design and identification of high-performance bimSACs.

## State-of-the-Art Theoretical Studies

### Density Functional Theory

Over the last few decades, density functional theory (DFT) has set a paradigm as one of the critical components in catalyst-development research to validate the catalytic mechanisms, understand fundamental chemical reactions, surface science, catalysis, and material science [116]. In the pursuit of novel materials, computational modeling by DFT calculations has offered deeper and more precise understanding of the reaction mechanisms [117]. DFT analysis can assist in calculating and discovering the whole reaction cycle and energy barriers of the single elementary step [118–120]. There is also very high practical value in the assessment of the electronic structure of the active sites and for unveiling structure–performance relationships as well as for the comprehensive study of complex materials [118, 119, 121]. DFT is also crucial for estimating the catalytic performance of SA engineered materials, together with their stability, activity, and selectivity [122]. High-throughput methods in DFT are based upon descriptors and are suitable for studying atomic-scale materials like SACs, including homo- and hetero-bimSACs (Fig. 5) [122]. In bimSACs, DFT studies play a crucial role in architecture prediction as well as interpretation of the intermetallic charge redistribution effects and intermetallic distances [83]. These predictive conclusions are considered as the standards for the evaluation of the accuracy of distinguished descriptors [83, 122]. Interpreting the influence of different structural units in the catalyst on its performance is crucial for development the next generation of SACs [120]. Xue et al. [123] applied DFT method to explore hydrogen dissociation over transition metal (TM = Ni, Pd) SAs dimers and trimers. Moreover, Guo et al. [124] utilized DFT calculations to obtain an activity map for nitrogen reduction reaction (N_2_RR) in bimSACs. The adsorption energy of N_2_H* was labeled as descriptor in the map, which was useful for the screening of promising bimSACs.

Kumar et al. employed DFT studies for evaluation of TM bimSACs stabilized on N-doped carbon (N–C) for HER according to the electronic states of the active catalytic centers [61]. The stability evaluation of TM bimSAC structures (heteronuclear: CoCu-SAD-N_6_C, NiCo-SAD-N_6_C, CoFe-SAD-N_6_C, CoMn-SAD-N_6_C; homonuclear: CuCu-SAD-N_6_C, NiNi-SAD-N_6_C, CoCo-SAD-N_6_C, FeFe-SAD-N_6_C, MnMn-SAD-N_6_C; where SAD stands for single-atom dimers) was carried out by estimation of formation energies (E_f_) [61]. The negative values of E_f_ obtained from the calculations revealed that the selected TM bimSACs were thermodynamically stable, demonstrating a cumulative trend with the number of outermost 3*d* orbital valence electrons. Interestingly, there was a consistent trend for the average Mulliken charges distribution (Δ*q*) of TM bimSACs centers with the formation energy of TM bimSACs structures, except for the case of CoCu/CuCu-SAD [61]. The elevated E_f_ with higher Δ*q* in the homo-/heterostructures of bimSACs suggested a thermodynamically more stable bimSAC structure [125]. Furthermore, the energy associated with water adsorption demonstrated a direct correlation with the Mulliken charge transfer from the metal active site. This indicated that a greater charge transfer from the active site results in stronger water adsorption capabilities [61]. A significant charge localization between the metal atom and the N coordination sites was confirmed with differential charge density distribution calculations [61]. Less electronegative TM atoms exhibited a higher tendency to donate electrons to N atoms and thus form stronger TM-N bonds [61]. Moreover, calculations of the partial DOS for numerous TM bimSACs confirmed that the *d*-orbitals of TM atoms were mostly distributed around the Fermi level [62]. It was also revealed that there was poor linear relation between the d-band center and formation energy; however, a linear correlation of the 3*d*-band center with the kinetic barrier of H_2_O dissociation was confirmed [61]. It was also revealed that the *d*-band centers of Co and Ni atoms in NiCo SAC dimers were the nearest to the Fermi level, indicating superior water dissociation ability and boosted proton adsorption, beneficial for HER [125]. Therefore, it was concluded that synergistic effects between Ni-Co bimSACs sites can stimulate water dissociation and efficiently adsorb the proton, which was proved crucial for achieving excellent HER activity based on the DFT studies [61]. By means of DFT calculations, Zhou et al. [126] investigated a series of two-dimensional (2D) homo- and heteroatom SACs on covalent organic framework (COF) material for OER. The designed 6 homonuclear (2TM-COF) and 15 heteronuclear (TM_1_TM_2_-COF) catalysts exhibited good stability [126], and a connection between the adsorption Gibbs free energies of HO* and HOO* intermediates was identified. The RhIr-COF exhibited the best OER catalytic activity with a *η*^OER^ value of 0.29 V, followed by the CoNi-COF system (0.33 V), RuRh-COF (0.34 V), and NiIr-COF (0.37 V) [126]. All the hetero-bimSACs exhibited lower onset potential and higher *j* compared to the benchmark catalyst IrO_2_(110) [126]. Assisted by the descriptor identification study, the Bader charge that is associated with the Pauling electronegativity of the implanted dual-metal atoms was found to be the most significant factor driving the catalytic activity toward the OER [126]. The balanced design of homo- and heteroatom catalyst system remains conceptually stimulating and involves in-depth research, both theoretically and experimentally [127]. Yang et al. [127] developed a polyoxometalate-based heteroatom SAC of O-coordinated W–Mo atoms embedded in N-doped graphene (W_1_Mo_1_-NG) (Fig. 6a) for electrocatalytic hydrogen evolution. The DFT geometry optimization indicated the bridging of O and W-Mo atoms anchored on NG vacancies through O_2_ atoms with W–O–Mo–O–C configuration [127], exhibiting potent active sites for HER, like noble metal-based catalysts. The electron delocalization on W–O–Mo–O–C motifs modulated the adsorption strength of H at medium levels (required for effective catalysis), thus boosting the HER kinetics, and overall performance. However, for homonuclear Mo_2_-NG and W_2_-NG models, atomic H adsorbed on the terminal O atoms linked with metal centers [127]. The metal-O_2_ bonds on the homonuclear SACs exhibited strong ionic nature, due to electrons partial localization to the O sites, leading to strong binding of H, and thus hampering dihydrogen formation [127]. These results suggested that the self-assembly of polyoxometalates can be a versatile method for formulation of effective dual-atom catalysts and particularly hetero-bimSACs [127].

**Fig. 4:**
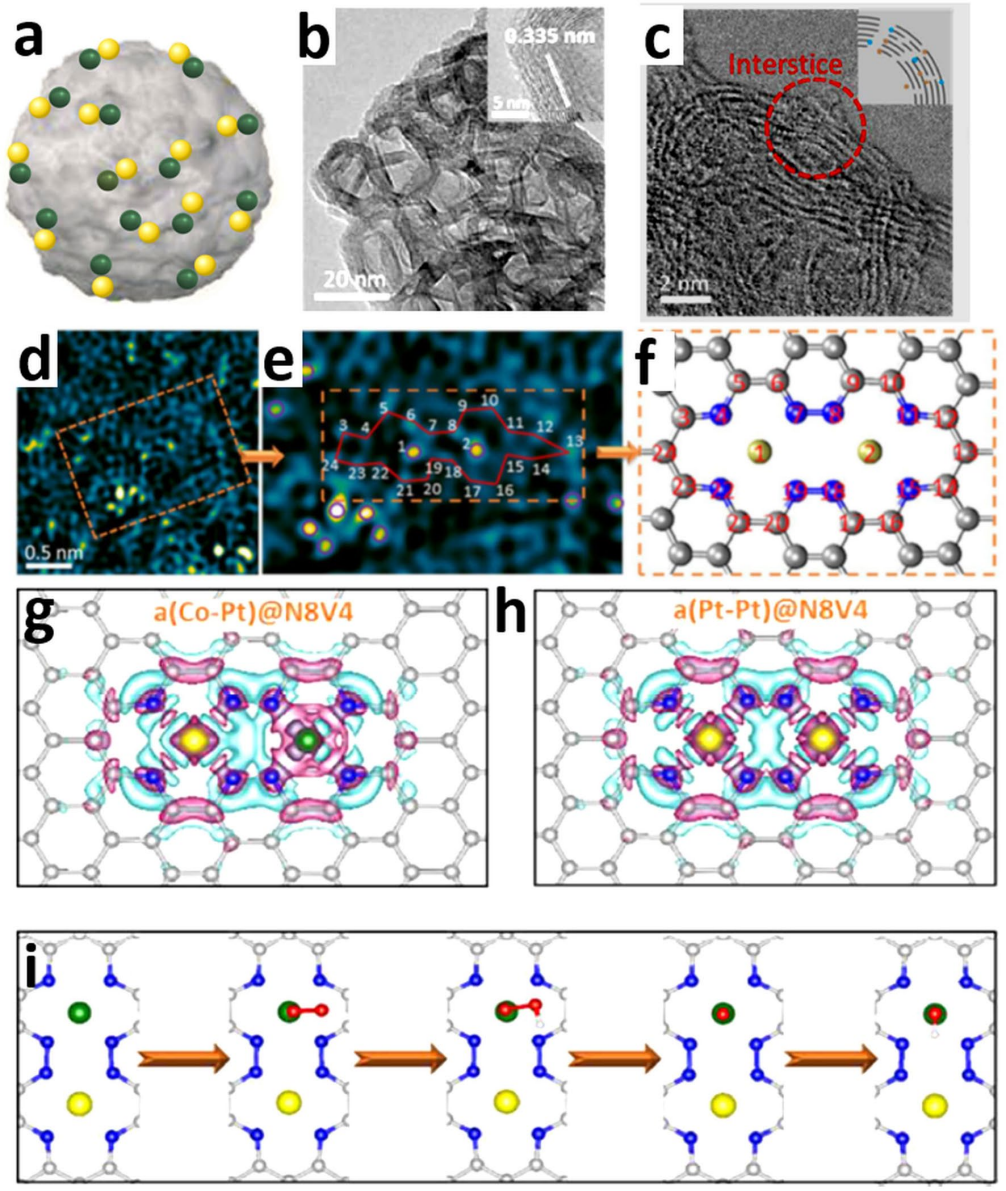
**a** Schematic of bimSAC structure. **b** TEM images of ACoPt-NC; the inset displays the hollow graphitic shells. **c** The bright-field STEM image of A-CoPt-NC. **d** The HAADF image of A-CoPt-NC after fast Fourier Transformation (FFT) filtering. **e** A partly zoomed-in image of the area mounted in (**d**), metal atoms are evident by purple circles. **f** Model of the configuration of the 2 metal atoms entrapped in the defect, recreated from the observed atomic structure in (**e**). **g**, **h** The top view of the charge densities of a(Co–Pt)@N8V4 (g) and a(Pt–Pt)@N8V4 (h). Pink and aqua isosurfaces with an isosurface level of 0.0025 e/a_0_^3^ signify electron accumulation and depletion areas, respectively. **i** Design of the ORR reaction pathway on a(Co–Pt)@N8V4. Reproduced with permission from Ref**.** [128]. Copyright 2018 American Chemical Society

Figure 4a indicates the structure of bimSACs and a locally dispersed atomic Pt–Co N–C-based catalyst (Fig. 4b, c) (denoted as A-CoPt-NC) showed remarkable selectivity for the four electron (4e^−^) pathway in ORR, differing from the reported 2e^−^ pathway typically observed in atomic Pt catalysts [128]. Figure 4d displays high-resolution transmission electron microscopy (HRTEM) analysis for the delineation of the local coordination environment of the metal atoms [128]. In Fig. 4e, the metal atoms are indicated with purple circles and their coordination environment is filled by the neighboring C/N atoms [128]. The structure observed in the electron microscope image is reconstructed in Fig. 4f. Such atomically precise knowledge of the coordination environment is of critical importance for the subsequent computational simulations [128]. The charge distribution patterns (Fig. 4g, h) revealed electron accumulation (pink area) around the Co atom in A(Co–Pt)@N8V4 (where N8 represents the number of N atoms and V4 indicates the number of vacant C atoms) type defect site, but poor electron accumulation around Pt in a similar site (Pt–Pt)@N8V4, which was ascribed to the asymmetric electronic distribution in the A(Co–Pt)@N8V4 motif, leading to substantial changes in surface charge at the catalytic active sites [128]. Figure 4i demonstrates the ORR mechanism associated with the 4e^−^ pathway on A(Co–Pt)@N8V4, which includes four protic hydrogen and electron transfer steps. DFT calculations indicated that this high activity originates from the synergistic effects of the atomic Pt–Co dimers positioned on the defected C/N graphene surface due to irregularity in the electron distribution around the Pt–Co metal dimers [128].

DFT has rapidly captivated the chemistry and materials science communities due to its versatile applications in catalysis research. Recognized as a foundational tool, DFT plays a crucial role in the development and analysis of catalysts. Numerous publications underscore its success, highlighting its extensive use particularly in materials chemistry for investigating molecular structures and reactions in both discrete molecules and extended systems. DFT has consistently demonstrated superior capability in accurately representing critical properties such as formation energies, atomic geometries, and charge densities, which are pivotal for computational studies in catalysis.

### Microkinetic Modeling

Microkinetic modeling is a key technique strengthening both experimental and theoretical observations and predicting the results of complex chemical reactions under various conditions. Microkinetic modeling is a computational approach used in chemistry and materials science to analyze and predict the kinetics of chemical reactions on a detailed level. It involves the identification of all relevant elementary steps in a reaction mechanism, such as adsorption, desorption, surface reactions, and diffusion processes, with each step having its own rate constant [129]. The rate constants are temperature dependent, taking also into account the coverage of reactants, intermediates, and products on catalytic surfaces, which can significantly influence the reaction kinetics [129]. Moreover, it is crucial for streamlining the development of bimetallic catalyst design, which relies on understanding the fundamental surface kinetics that control catalyst performance [130]. Microkinetic modeling plays a significant role in identifying critical reaction intermediates and rate-determining elementary reactions and is thereby vital for designing improved catalysts [131]. Heterogeneous catalytic transformations of small molecules can be investigated more precisely via combining microkinetic modeling with DFT [130]. Microkinetic modeling, enhanced by DFT energies, is crucial for understanding the fundamental chemistry of catalytic reactions and linking theoretical and experimental insights [132]. It involves creating a series of ordinary differential equations based on thermodynamic properties and kinetic parameters of reaction steps [132]. Catalysis is a kinetic phenomenon, and chemical kinetics are thus important in catalysis research. Reaction kinetics data are used as key ingredient in reactor design, to analyze reaction mechanism and illuminate the structure–property association of a catalyst. Efficient explanation of reaction mechanisms along with surface chemistry can offer significant insights for the rapid development of catalysts with better performance [130]. Accurate microkinetic modeling requires consideration of surface coverage effects, which significantly influence reaction rates and selectivity. Therefore, there is a need for a more comprehensive understanding and inclusion of these effects in modeling. Such microkinetic calculations are principally ab initio calculations, allowing to reproduce and predict macroscopic reaction kinetic results under experimental conditions, while delivering thorough and quantifiable conclusions about reaction mechanisms [132]. Accurate first-principles determination of adsorption and desorption processes is critical for refining model predictions and achieving theory–experiment correlation. The development of advanced microkinetic models can more accurately reproduce experimental observations and guide the development of more effective catalysts.

In electrocatalytic applications, the demand for high-performance electrocatalysts that enable energy-efficient and environmentally friendly electrochemical conversions has significantly heightened research interest over the past few decades [133]. Understanding the influence of rate-controlling reactions and the effects of transitional surface intermediates on the overall reaction kinetics is crucial for designing the next generation of efficient electrocatalysts [134].

### Ab Initio Molecular Dynamic (AIMD) Simulations

The intricate details of chemical processes in solid phases have garnered significant interest in contemporary theoretical research, boosted by the advancements in high-speed computing [135]. Molecular dynamics (MD) stands out as a pivotal theoretical technique for these investigations. It not only provides insights into both the equilibrium thermodynamic properties and the dynamics of systems at a fixed temperature, but also offers a microscopic view of the motion of individual atoms within the system [136]. Additionally, this model is typically parameterized by fitting to experimental data or high-level *ab* initio calculations on small clusters, establishing it as one of the most successful methodologies for analyzing diverse systems ranging from simple liquids and solids to polymers and biological entities [131, 137]. Despite its remarkable efficacy, the conventional force field approach in MD faces significant limitations [137]. Firstly, charges are treated as static parameters, hence neglecting electronic polarization effects. Secondly, force fields generally assume fixed atomic interactions, thereby lacking the capability to accurately depict chemical bond dissociation and formation [135]. These constraints can be addressed by one of the most noteworthy recent advancements in MD related to ab initio molecular dynamics (AIMD) methods [137].

AIMD simulation methods employ quantum mechanical calculations to predict electronic structure [138]. To accurately model and replicate the full spectrum of catalytic effects induced by electric fields, AIMD simulations are essential. These simulations offer unique capabilities of managing multiple types of covalent bond rearrangements, making them particularly suitable for understanding chemical reactions [139]. AIMD simulations compute forces based on electronic structure calculations executed throughout a simulation and can reliably model the dynamic association of solvent molecules. Consequently, AIMD can elucidate the role of specific molecules within the solvent environment and is employed to model liquid-phase reactions [140]. Furthermore, AIMD, when combined with advanced sampling techniques, enables more effective investigation of chemical phenomena in catalytic systems [141]. These techniques incorporate crucial parameters such as fixed temperature effects and cooperative dynamics, providing robust data on enthalpic and entropic contributions that significantly affect the reaction free energy profiles [141]. This approach contrasts with traditional ab initio static methods, which rely on calculating reaction free energies from various coarse-grained models of the reaction potential energy surface. First-principles simulations, especially of increasing complexity like solid/liquid catalytic interfaces, are effectively conducted using enhanced sampling with AIMD [137–139]. AIMD supports a systematic approach toward the construction of efficient electrocatalysts for energy production by predicting and tuning the energies of reaction intermediates and kinetic barriers along with fine-tuning reaction conditions.

### Machine Learning

**Fig. 5:**
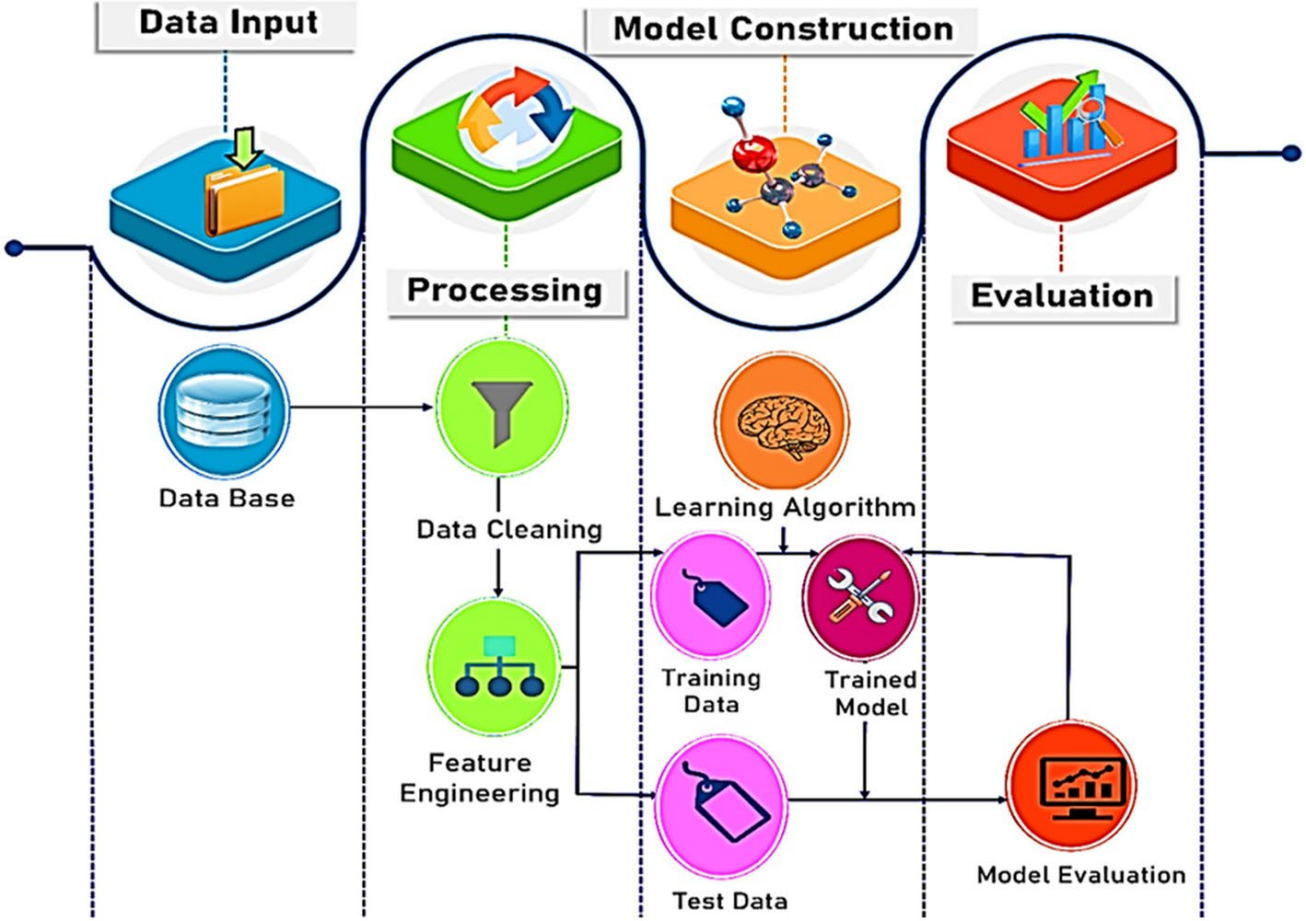
Machine learning roadmap. Reproduced with permission from Ref. [142]. Copyright 2022 Wiley–VCH GmbH

Machine learning (ML) is an important aspect of artificial intelligence which has received remarkable attention in numerous fields of science and technology for the development of algorithms and software for quantitative understanding, language processing, image identification, and for making fast and precise predictions [72]. The building blocks of ML workflow are based upon the existing data and carefully chosen algorithms, to constantly improve models, and suggest promising directions, as shown in Fig. 5 [142]. ML is an interdisciplinary field of study within computer science, statistics and numerous subjects in data science. It signifies wide acceptability and potential in resolving future scientific challenges [72]. However, application of ML in the field of catalysis is in its early stage of development. Conventionally, catalyst design and synthesis critically rely upon trial and error with chemical intuition, which is time-consuming and cost-demanding [72]. It has been understood that automated ML processes are crucial in building better models, to understand the catalytic mechanisms, and provide an insight into novel catalytic design [142]. This approach can be further supported by the development of state-of-the-art algorithms and theory, easy accessibility of experimental data, as well as offering the benefit of low computational cost.

ML approaches are broadly classified into supervised and unsupervised learning based on specific tasks employed for resolving practical problems [142]. Supervised learning methods are of much practical value in deep learning for discovering predictive models. Linear regression, artificial neural networks (ANNs), support vector machines (SVMs), and random forests represent diverse machine learning (ML) methodologies. Each is based on distinct algorithms related to various regression and classification tasks. ML methods have their corresponding advantages and disadvantages, and the choice of specific method depends on the size and features of the database [142]. Linear regression and classification methods can handle small data sets, including Logistic and Naive Bayes methods [143]. On the other hand, nonlinear methods are desirable for large data sets, such as ANN and k-nearest neighbor (KNN) methods. Besides, there are also approaches that can be employed for both linear and nonlinear analyses, such as SVM, which is simpler to understand than ANN, but it is limited by its sensitivity to the selection of parameters and functions [144]. SVM studies need to be performed by expert users; therefore, the property of SVM models varies according to the appropriate setting of a substantial number of parameters [145]. Furthermore, SVM algorithms need excessive computational resources owing to the nature of their optimization problem [145]. Therefore, the least squares support vector machines (LS-SVM) method is proposed, which represents a variation in the traditional SVM algorithm [146].

For homogeneous catalysts, linear regression analysis is widely reported to create a quantifiable association between the structural descriptors and catalytic activity and/or other properties [72, 147]. Heterogeneous catalysis involves the interface of a molecule with a substrate, offering attractive features [72, 147]. In heterogeneous catalysis, the data set is from constant processes which provides the surroundings for the variation in a limited number of parameters and makes it effortless to directly create a vast data set [72, 147]. Therefore, there are more reports on ML-related studies in the field of heterogeneous catalysis compared with those in homogeneous catalysis [72, 147]. The association of ML with quantum mechanics (QM) calculations has inspired researchers to enhance the discovery of catalyst candidates in combinatorial extended spaces, such as bimetallic alloys [148]. High-level quantum–chemical calculations are accurate in delivering reactivity descriptors, but the high computational costs limit broad application [148]. On the contrary, the ML methods are promising alternatives to model the reactivity of catalysts based on the association between structural descriptors and reactivity properties [72].

An ML methodology employing the gradient boosting regressor (GBR) algorithm was employed to calculate the binding energies of oxygen (E_O_) and carbon (E_C_) atoms on SAA of Cu, Ag, and Au [147]. The periodic properties of the TMs function as input elements in the model [149]. Their influence in adsorbate–metal interaction was evaluated toward building a systematic descriptor. In test runs, the ML model could predict E_O_ and E_C_ with considerably low error (∼0.2 eV) [149]. Lu et al. developed ML models trained with (Density Functional Theory) DFT calculations to predict the thermodynamic stability of SA and their associated coordination site of 38 different elements alloyed with Cu [150]. A Gaussian process regression (GPR) model achieved the best results with a mean absolute error (MAE) of less than 0.08 eV for accumulation energy. Similar performance was attained with an even smaller training dataset employing an active learning algorithm, delivering ca. 35% time saving. Furthermore, the ML model was applicable to several other substrates (in addition to Cu), different adsorbates (in addition to O*), and bigger cluster sizes (greater than trimers), exhibiting the potential to address a large number of degrees of freedom while substantially saving of time [150].

ML is pivotal for the theoretical analysis of bimetallic catalysts, as it helps unveil the structures and reactions within complex analytical systems. Thus, there is a marked trend in catalysis research toward employing ML toward this direction. Advanced methodologies developed by research communities are elucidating the intricate structures and reaction networks by integrating ML potentials with effective and comprehensive optimization systems. The high precision and rapid processing capabilities of ML techniques are driving the development of innovative algorithms that may address long-standing challenges in bimetallic catalysis, thereby unlocking significant potential for developments in the field.

## Physicochemical Characterization Techniques

Successful synthesis of SACs is particularly challenging including their precise characterization and description of their coordination environment. Until now, quite a broad portfolio of state-of-the-art techniques has been utilized to confirm the presence of SAs, their valence state, and local coordination, such as aberration-corrected high-resolution STEM (particularly when used with detectors like the HAADF), electron energy loss spectroscopy (EELS), or (XAS). Recent research findings increasingly recognize that catalysts are dynamic systems that actively adapt and respond to their reaction environments, rather than being merely static arrangements of atoms. This paradigm shift necessitates more sophisticated experimental tools, enabling precise probing of the composition, structure, and dynamics of catalysts under authentic reaction conditions. In situ and operando techniques have advantages in terms of providing information on the state of SAs under turnover conditions, since the atomic structure of SACs might undergo changes during the interaction with the substrate or intermediates [151]. Such advanced methods have been successfully applied both in thermal-, photo-, and electrocatalysis [152].

### X-Ray Absorption Spectroscopy

X-ray absorption spectroscopy (XAS) provides critical insights into the presence of SAs and has emerged as an essential tool for investigating the structure and composition of bimSACs. This technique elucidates the local geometry and electronic structure of catalysts by offering element-specific analysis. It is particularly effective in probing active sites within multi-element systems and is versatile enough to examine a diverse array of materials, including ordered and disordered solids, nanostructures, and liquids [152]. In case of surface-anchored atoms, XAS is an extremely sensitive technique and thus appropriate even for probing catalysts across the periodic table at very low concentrations [153]. In addition, XAS is able to examine variations in 3D, 4D, and time-resolved regimes during function and can explore materials in different phases like amorphous, crystalline, and homogeneous liquid [153]. When it comes to dealing with bimetallic catalysis and specific in SACs, XAS is more beneficial than traditional techniques to explore the actual structure of unique materials [153]. XAS has already proved its expertise in bimetallic catalysis study. First, the measurement selectively focuses on the element of relevance devoid of intervention from additional elements. The measurement potential of XAS is independent of crystallinity, isotope labeling, etc. with advantage of low concentration limit (about 50 ppm) appropriate for liquid-phase-supported catalysts under broad range (− 269–1200 °C) under conditions from vacuum to high pressure.

**Fig. 6:**
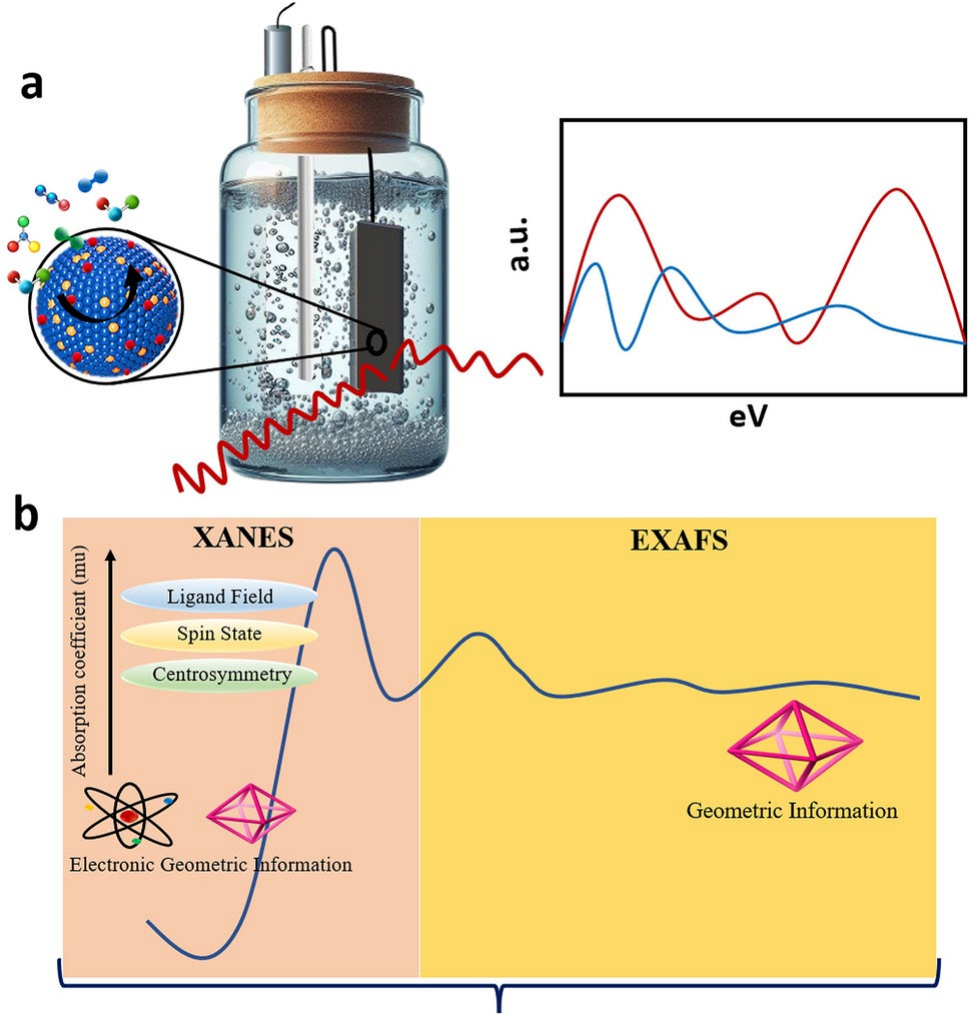
**a** Experimental setup for in situ X-ray absorption spectroscopy. **b** Typical spectrum of XAS measurement illustration for different regions

Since few decades, in situ XAS has been dedicated to monitor the intrinsic properties of materials during catalytic turnover, including physical and electrochemical tests [154]. In in situ XAS measurements, electrochemical tests have propelled rapid advancements in combining XAS spectrometer [154] and three-electrode cell (Fig. 6a) [155]. The efficient blend of synchrotron radiation and superior penetration ability of high-energy X-rays essentially offers atomic-level information of the processes taking place during the electrochemical reactions [156]. On the other hand, soft X-ray-based in situ XAS helps to discover the environment of O_2_ atoms in materials [157]. The X-ray absorption near-edge structures (XANES) and the extended XAFS (EXAFS) are the two parts of XAS (Fig. 6b) used under reaction conditions with synchrotron radiation [158–161]. XANES displays the electron and oxidation states of catalysts while EXAFS offers evidence of chemical bonding, interatomic distance, and coordination number of catalysts [158–161]. XANES is correlated with the region -50 to 150 eV from the absorption edge to study the electronic transitions taking place in inner shells up to valence orbitals following the dipole selection rule (Δl =  ± 1). XANES serves as an essential tool to uncover the structural evidence involving oxidation and electronic states, magnetic properties, and geometry of materials’ structure [161], while offering the advantage of being an element-selective technique [162]. The electronic information in XANES spectrum is evident in different forms. The relative position of the absorption edge shows the actual charge on the absorbing atom; the location and intensity of the resolvable features offer further information about the relative width and occupancy of the electronic states [163]. Additionally, the polarized environment of the synchrotron radiation offers additional evidence in the study of the anisotropy of the final electronic states in single crystals or oriented samples [163]. When the X-ray photon energy (E) is regulated to the binding energy of core level of an atom in the material, sudden raise in the absorption coefficient, known as the absorption edge, takes place [164]. For isolated atoms, the absorption coefficient reduces continuously as a function of energy beyond the edge. For atoms both in a molecule or embedded in a condensed phase, the variation in absorption coefficient at energies above the absorption edge displays a complex fine structure called EXAFS [164]. EXAFS entails the oscillatory variation in X-ray absorption as a function of photon energy beyond the absorption edge [164]. The absorption, usually stated in terms of absorption coefficient (μ), is evaluated from a measurement of the attenuation of X-rays upon their passage through a material [164].

**Fig. 7:**
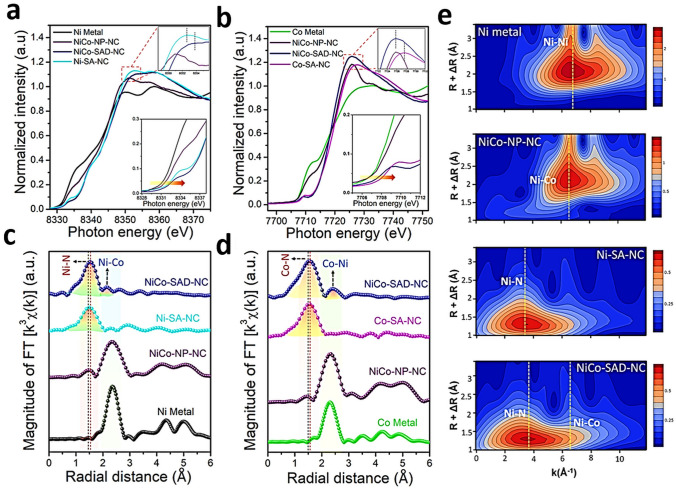
**a** Ni K-edge and **b** Co K-edge XANES spectra of NiCo-SAD-NC with reference samples. **c** Ni K-edge, and **d** Co K-edge FT-EXAFS spectra of NiCo-SAD-NC with reference samples. **e** WT-EXAFS of NiCo-SAD-NC with reference samples at Ni K-edge. Reproduced with permission from Ref***.*** [61] Copyright 2021 Springer Nature

To investigate the electronic states and local coordination chemistry of Ni/Co SAD as catalyst XPS, XANES and EXAFS measurements were carried out [61]. After bimetallic NiCo formation, from the fitting of the Ni K-edge XANES energy at half-edge jump, and fitting of the Ni 2*p*_3/2_ XPS spectra of NiCo-SAD-NC, nickel was estimated with a more positive oxidation state of + 1.73 eV compared to that of Ni in Ni-SA-NC (+ 1.57), whereas the Co 2*p*_3/2_ XPS spectra of NiCo-SAD-NC displayed a negative shift with Co oxidation state of + 1.39 compared to Co in Co-SA-NC (+ 1.67) [61]. These observations suggested the electron transfer from Ni to Co site at the atomic interface of NiCo-SAD, through the single Ni-Co bond formation at the atomic level stabilized by N coordination [61]. In XANES spectra of NiCo-SAD-NC, the near edge and white line features in the Ni K-edge showed positive shift, revealing a higher oxidation state of Ni in NiCo-SAD-NC compared to Ni-SA-NC and matched well with Ni 2*p* XPS results (Fig. 7a) [31]. The pre-edge peak which appeared around 7709.5 eV for NiCo-SAD-NC, Co-SA-NC and for the standard cobalt phthalocyanine in the Co K-edge profile, suggested the presence of X-ray absorbing Co centers with four coordination (N or metal, Fig. 7b) [61]. Remarkably, the typical Ni–N bond length was efficiently shifted for NiCo-SAD-NC compared to Ni-SA-NC, demonstrating a distorted *D*_4h_ local symmetry of Ni atom site with the simultaneous appearance of Ni-metal peak at 2.18 Å that was absent in Ni-SA-NC, supporting the in-situ formation of extra Ni-Co coordination along with Ni–N bonds, in agreement with the previously reported Zn-Co and Co-Fe dual sites [165, 166] (Fig. 7c). Furthermore, in the Co K-edge FT-EXAFS spectra, the extension of Co–N bond from 1.48 Å (the case of Co-SA-NC) to 1.56 Å (for the case of NiCo-SAD-NC) advocated the distorted *D*_4h_ local symmetry of Co atom center with the development of an extra Co–Ni bond with a length of 2.40 Å, which was absent in Co–SA–NC, supporting the presence of NiCo dimer structure (Fig. 7d) [61].

The complex nature of heterogeneous catalysts poses challenges for the design of novel catalytic materials [167]. However, the precise nature of SAA catalysts has enabled the application of well-defined theoretical models combined with accurate surface science experiments. For example, Hannagan et al. reported the theory-led discovery of RhCu SAA [167]. The RhCu/SiO_2_ SAA catalyst was placed at high temperature in situ at 673 K under hydrogen for 1 h and further cooled at room temperature and He was purged. The spectra were recorded in fluorescence mode under He flow at room temperature. The successful RhCu SAA structure formation without surface aggregates was confirmed by in situ EXAFS studies [167]. Giulimondi et al. [168] reported Au-Ru dimers, as substantiated by detailed XAS analysis. Precisely, the wavelet transforms investigation of the EXAFS spectra evidenced to be a decisive tool for the assessment of intermetallic bonds in heavy adjacent atoms, distinguishing scattering pairs at similar distances that would be difficult to resolve entirely by the frequently used Fourier transform analysis [168]. The electronic fingerprints of the two configurations were investigated by means of spectroscopic techniques, unveiling electron density transfer from Ru to Au, which was responsible for a medium strength proton adsorption, leading to improved catalytic features in HER [168]. Similarly, to unravel the origin of the cooperativity of bimetallic Ru-Pt SACs, the bimSAC structure was comprehensively examined by XAS analyses [83]. The careful XAS study of Pt-Ru dimers confirmed the direct bonding between Pt-Ru [103]. First-principles calculations showed that the Pt-Ru dimer creates a cooperative effect by regulating the electronic structure, which results in the superior hydrogen evolution activity [103].

### High-Angle Annular Dark-Field Scanning Transmission Electron Microscopy

**Fig. 8:**
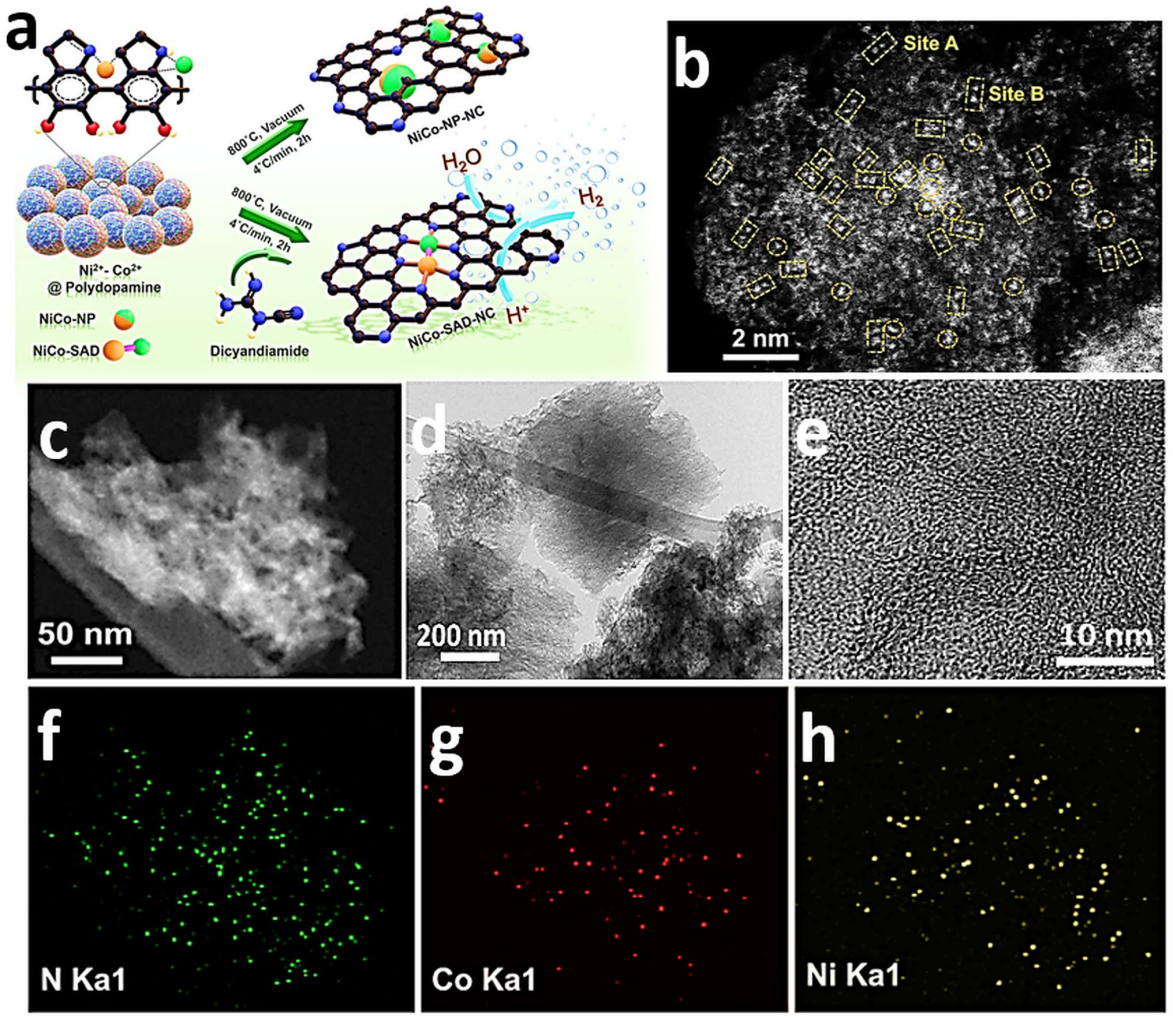
**a** Schematic of the synthetic strategy for the NiCo-SAD-NC and NiCo-NP-NC. **b** Aberration-corrected HAADF-STEM image of the NiCo-SAD-NC. (The yellow square in **b** indicates the dimer sites and orange circles shows the single Ni/Co atom sites.) **c** HAADF-STEM and **d, e** TEM, HRTEM image of NiCo-SAD-NC. Corresponding energy dispersive X-ray spectrometry (EDS) maps of NiCo-SAD-NC illustrating the consistent dispersion of **f** N (green), **g** Co (red), and **h** Ni (yellow). Reproduced with permission from Ref. [61]. Copyright 2021 Springer Nature. (Color figure online)

**Fig. 9:**
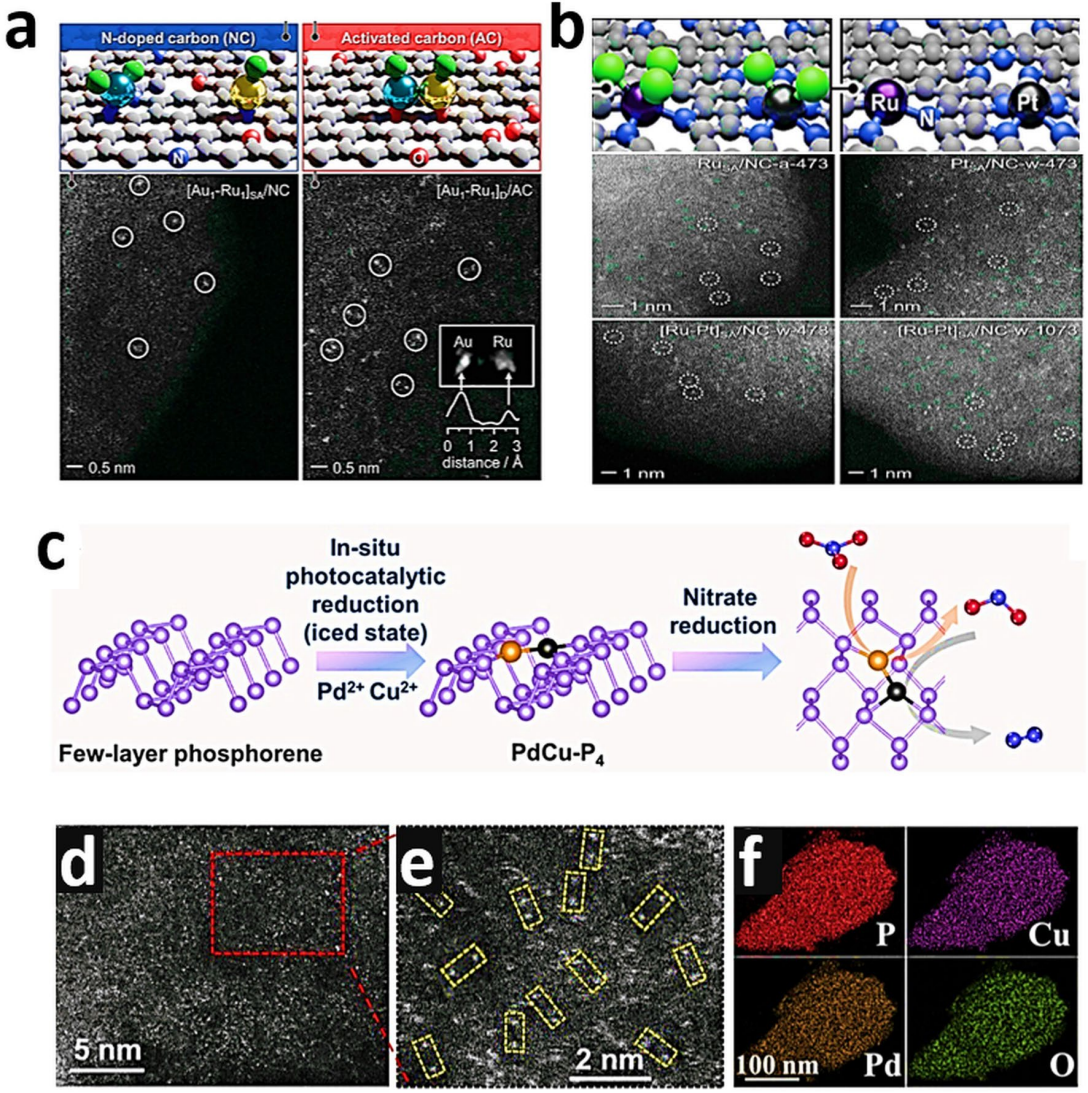
**a** Schematic representation of the dimers and spatially isolated atoms in bimetallic Au-Ru catalysts via C-host functionalization. Reproduced with permission from Ref. [168]. Copyright 2022 Wiley–VCH GmbH. **b** Bimetallic Ru*-*Pt single-atom catalyst along with HAADF-STEM images, respectively. Reproduced with permission from Ref. [83]. Copyright 2022 Wiley–VCH GmbH. **c** Schematic pathway of ultrathin PdCu-P_4_. **d, e** HAADF-STEM images, and **f** EDS mapping of PdCu-P_4_ cathode. Reproduced with permission from Ref. [176] Copyright 2022 Wiley–VCH GmbH

High-angle annular dark-field scanning transmission electron microscopy (HAADF-STEM) is key analytical technique to study with atomic resolution SACs. This technique is sensitive to the atomic number (Z) [169], facilitating the identification of individual foreign atoms residing inside the crystals and various solid supports [169]. In HAADF, the atomic columns along with the dynamical diffraction of the probe enhance the column intensities, making them sensitive to the presence of an impurity atom [170]. Individual atoms are identified by the appearance of bright spots in the image. Materials with higher atomic numbers scatter a larger number of electrons, resulting in brighter spots in the image [171]. With the help of Z contrast and aberration correction, heavy elements are easy to visualize at the atomic scale, providing evidence of the existence of SAs. SAs [172], dual atoms, and alloys of atoms can all be precisely distinguished via intensity profiles [173]. More precisely, heavy metal SAs on crystalline supports or low atomic weight supports are easy to capture, with the Z contrast being crucial for the identification of the single heavy atom with a higher intensity. Straightforward visualization of SAs offers a clear proof of the successful synthesis of single-atom-engineered materials [171]. Ro et al. reported the existence of different metal combination-based dimers by HAADF-STEM using the intensity difference to demonstrate the successful formation of co-localized atomically dispersed Rh and Re dimers [174]. Kumar et al. [61] reported bimSAC interfaces for hydrogen evolution. A facile synthesis strategy to obtain NiCo-bimSAC was illustrated by precise control of N moieties (Fig. 8a). The aberration-corrected HAADF-STEM image in Fig. 8b revealed the uniform presence of isolated Ni-Co bimetallic sites (marked by the yellow squares) with coordination between Ni and Co along with some isolated Ni or Co atoms (marked by the orange circles) [61]. Moreover, HAADF-STEM (Fig. 8c) and HRTEM (Fig. 8d, e) images suggest the atomic dispersion of Ni and CO species in the NiCo-SAD. Corresponding EDS elemental mapping showed that N, Ni, and Co atoms were homogeneously distributed in the NiCo bimSAC and did not exist in the form of NPs, aggregations, or clusters (Fig. 8c–f) [61]. Giulimondi et al. also reported bimetallic Au-Ru catalysts [168] and bimetallic Ru-Pt SACs (Fig. 9a, b) [83], where the atomic dispersion was visualized by HAADF-STEM imaging. These results demonstrated that the integration of stable single-metal atoms in SAC synthetic procedures is a viable and valuable strategy to control the nuclearity in sintering-prone metal atoms (e.g., Ru atoms) and attain distinct coordination properties compared to their monometallic counterparts.

The combination of HAADF-STEM with energy dispersive X-ray (EDX) elemental mapping can be particularly helpful to reach the milestone toward more detailed imaging of complex heterostructures comprised of two metal elements with similar atomic numbers [175]. Sun et al. [176] reported 2D phosphorene as a giant P ligand to constrain high-density Pd–Cu dual-atom system to create an exceptional PdCuP_4_ coordination structure (Fig. 9c). Spherical aberration-corrected atomic resolution HAADF-STEM confirmed that the metals were atomically dispersed, and the pairs of bright dots are Cu − Pd pairs (Fig. 9d, e) [176]. EDS mapping showed that P, Pd, and Cu elements are uniformly allocated (Fig. 9f) [176]. Therefore, HAADF-STEM studies provide direct evidence through visualization of catalysts at the atomic level with less high accuracy in localizing atomic positions compared to conventional microscopic techniques.

### Surface-Enhanced Raman Spectroscopy

Surface-enhanced Raman scattering (SERS) is a widely used technique that exploits the inelastic scattering of light by molecules. It has the potential to detect single molecules under certain conditions, particularly when the molecules are adsorbed onto nanostructured surfaces, such as those made from silver or gold [177]. The exclusive vibrational spectroscopic information offered by SERS differentiates it from other conventional techniques, facilitating its application in multi-dimensional ways across electrochemistry, catalysis, biology, materials science, and others [177]. SERS can be attained from the electric field (EF) enhancement at the surface of single NPs, but also by using more intricate structures, for instance, by positioning the molecules within a few nanometer-sized gaps between two metal particles (so-called hotspots), which facilitate intense EFs as large as EF ≈ 10^5^–10^6^ V m^−1^ [178–181]. SERS is a critical technique for analytical applications as it significantly increases the inherently low efficiency of conventional Raman scattering. The typical Raman scattering cross-section (dσ_R_/dΩ ~ 10^–31^ cm^2^ sr^−1^) is considerably lower compared to the cross-sections for fluorescence emission (dσ_F_/dΩ ~ 10^–16^ cm^2^ sr^−1^) and infrared absorption (dσ_IR_/dΩ ~ 10^–20^ cm^2^ sr^−1^), respectively [182]. Therefore, SERS enables the analysis of even single molecules [182]. Monitoring the structure and behavior of single molecules is of great interest in various fields [183]. Single-molecule SERS detects the vibrational modes of single molecules, delivering high throughput structural information [183]. Ma et al. reported atomically dispersed binary Ni_x_Fe_100−*x*_-NC (x = 0–100) materials with tunable Ni/Fe ratios. Upon studying these materials, strong synergy was revealed as a crucial factor for the improvement in ORR and OER [184]. I*n situ* Raman characterization confirmed that the Fe site in Ni(N_3_)-Fe(N_3_)-C_n_ motifs was the accountable site for both ORR and OER [184]. Therefore, SERS can be employed as a sophisticated vibrational spectroscopic technique that is sensitive enough to probe individual atoms and provides fine molecular fingerprints that allow direct identification of target molecules.

### Electron Energy Loss Spectroscopy

Electron energy loss spectroscopy (EELS) is a powerful analytical technique primarily used in materials science and condensed matter physics which measures the energy loss of electrons as they pass through a material, providing detailed information about the electronic structure and composition. The technique is typically implemented in conjunction with a TEM [185]. Recent advancements in aberration correctors and monochromators offer remarkably high spatial resolution and high energy resolution [185, 186]. The combination of aberration-corrected optics, pixel array detectors, and full-field ptychography allows the spatial resolution can extend to 0.039 nm [187]. EELS, when combined with STEM, offers chemical information at atomic scale [185] and provides an alternative method of elemental analysis in TEM, especially for elements of low atomic number [188]. EELS is also capable to explore the local density of unoccupied states at sub-nanometer spatial resolution, offering a potent means to chemically analyze the composition of a material [189].

**Fig. 10:**
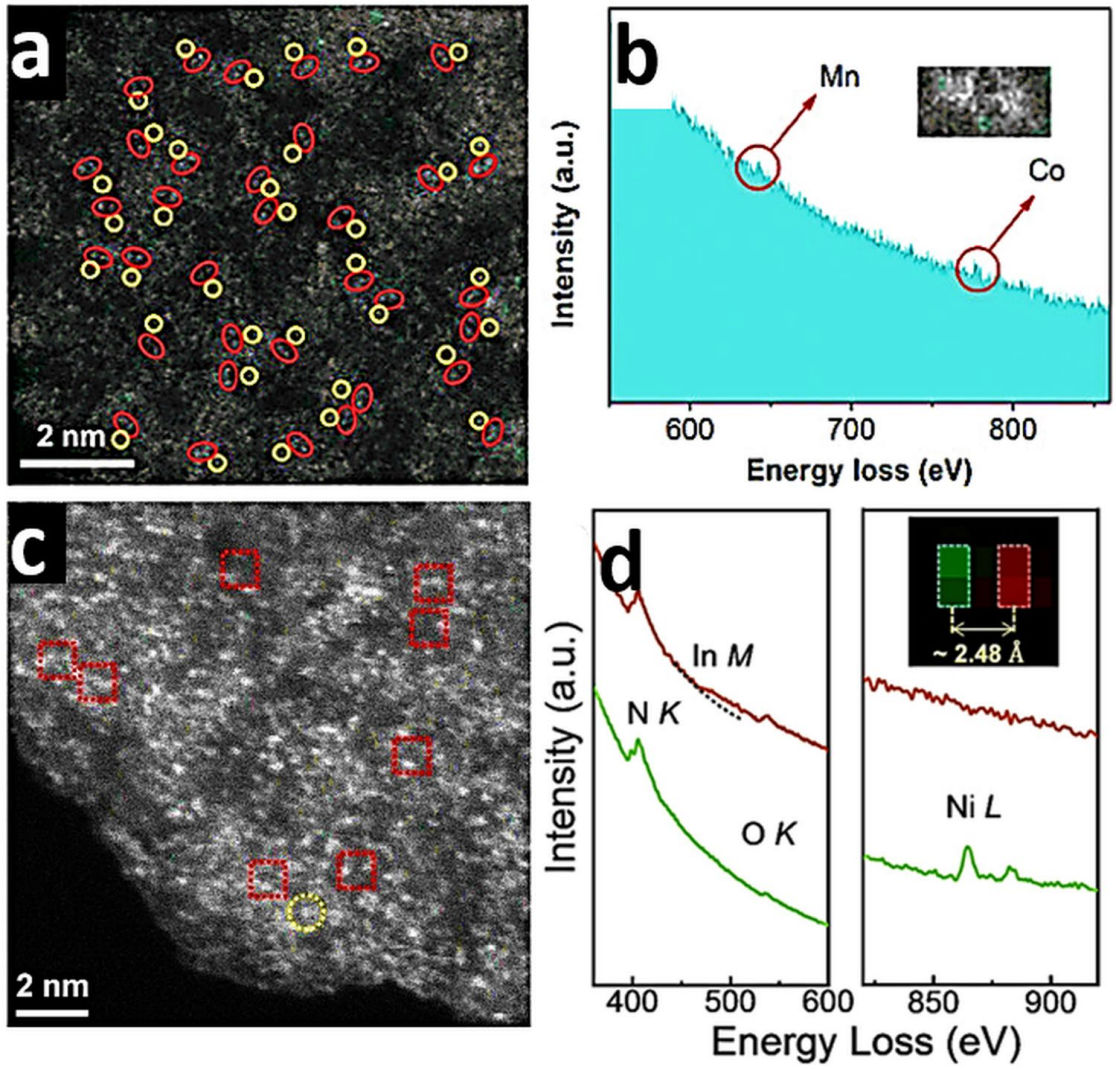
**a** Aberration-corrected HAADF-STEM image of Co/CoMn-NC (Co-Mn dual sites are marked by the red circles). **b** Co/CoMn-NC structure analyzed by EELS. Reproduced with permission from Ref. [198]. Copyright 2024 John Wiley and Sons**. c** Aberration-corrected HAADF-STEM image of InNi DS/NC. **d** The EELS spectra extracted for the InNi DS/NC. Reproduced with permission from Ref. [199] Copyright 2024 John Wiley and Sons

In bimSACs, the combination of different metal species results in distinctive characteristic peaks in EELS distinguished by their differing energy positions [190]. Hetero-bimSACs can be categorized into two types based on the difference in the atomic numbers of the constituent elements [191]. In the first case, **dual atom pairs with significant atomic number differences** can be easily identified using aberration-corrected HAADF-STEM due to their distinct Z contrast [192]. An example of this type is the FeRu bimSACs, where the large difference in atomic numbers between Fe and Ru makes them easily distinguishable in aberration-corrected HAADF-STEM images [193]. In the case of **dual atom pairs with close atomic numbers**, it is difficult to differentiate using HAADF-STEM because of their very similar intensity profiles [194]. In such cases, EELS can be employed to confirm the composition of the metal atom pairs. An example of this type is the case of the CoFe bimSACs [193]. While the existence of FeFe and CoCo bimSACs cannot be completely ruled out, EELS analysis of eight different randomly selected sites primarily indicated the presence of CoFe dual-atom pairs [193]. Zhang et al. designed a Ni-Cu bimSAC dispersed on hollow N–C for CO_2_ electroreduction [195]. The strong electronic interaction between the Ni and Cu atoms suggested a potential coupling or correlation of these atoms. EELS confirmed the coexistence of Ni and Cu atoms within the bimSAC structure. The presence of neighboring bright dots with a distance estimated to be 2.6–2.7 Å suggested a strong electronic interaction between the two atoms, possibly implying the coupling of Cu and Ni atoms. The high-resolution XPS Cu 2*p* spectra and Ni 2*p* spectra showed shifts in binding energy, indicating electronic interactions. The Cu 2*p*_3/2_ peak of Cu/Ni-NC was located between Cu(I) and Cu(II), while the Ni 2*p*_3/2_ peak of Cu/Ni-NC shifted to lower binding energy compared to Ni-NC. These shifts suggested that the Cu and Ni atoms were influencing each other's electronic states, supporting the EELS findings [195]. Wang et al. developed a host–guest strategy to produce a Fe-Co hetero-bimSAC electrocatalyst embedded on N–C for ORR [196]. The abundant formation of Fe–Co binuclear sites within the C scaffold was confirmed by EELS mapping [196]. Gu et al. utilized EELS elemental mapping and other spectroscopic techniques to identify Ni and Cu atoms and elucidate their structural configurations in a Ni_1_Cu_2_ trimer anchored on a g-C_3_N_4_ support [197]. Sun et al. [198] introduced CoMn bimSACs on a N–C substrate (Fig. 10a). The actual presence and identity of the Co and Mn atoms were confirmed using EELS (Fig. 10b), which verified the intensity profile and the corresponding electron energy loss peaks. An atomic indium–nickel dual-site catalyst was synthesized on nitrogenated ZIF-derived C (InNi DS/NC) support where the metals were coordinated on the graphene plane of the support and an axial O_2_ atom connected the metals via an out-of-plane bridge (O-In-N_6_-Ni moiety) [199]. The diatomic configuration and coordination of the catalyst were confirmed by AC HADDF-STEM and EELS (Fig. 10c, d) [199]. K-edges of N and O_2_ were detected in the vicinity of the metal atoms, suggesting a mixed N/O coordination environment for the metals. EELS is a highly effective technique for investigating the electronic structure of active sites on catalysts. It offers exceptional sensitivity in detecting and mapping the distribution of both light elements, such as C and TMs [200]. EELS excels in providing detailed insights into the atomic-scale composition and electronic states of these materials [200]. In recent years, EELS has become increasingly significant for characterizing the presence and distribution of SACs and bimSACs on various supports [172, 194, 199]. Its ability to precisely identify and differentiate between different atomic species, even those with similar atomic numbers, makes it a key tool for confirming the existence and structure of hetero-bimSACs [193]. By providing both spatial and chemical information at the atomic level, EELS contributes significantly to the understanding of how these dual-metal sites interact and function within catalytic systems.

## BimSACs in Catalytic Applications

### Hydrogen Evolution Reaction

**Fig. 11:**
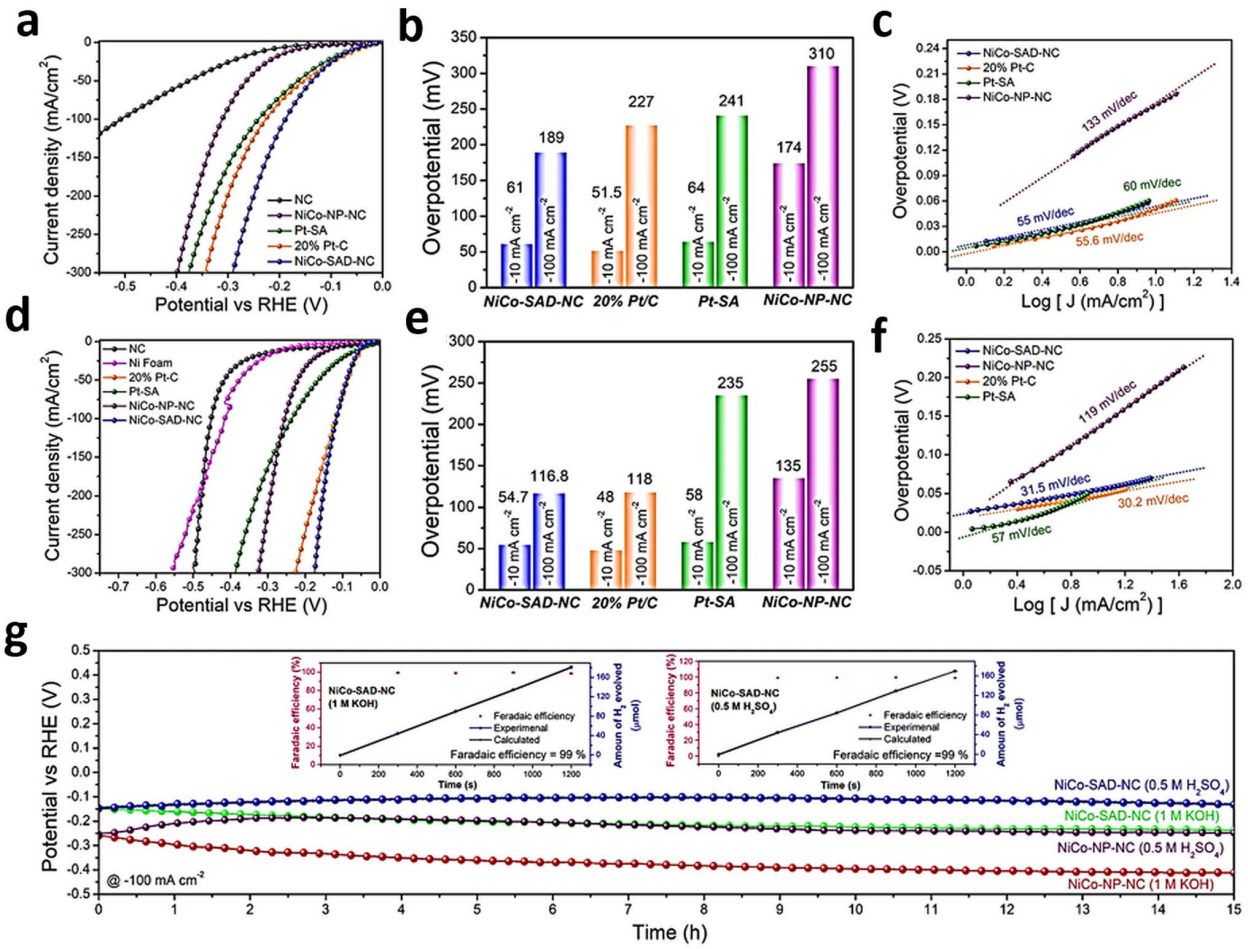
**a** HER linear sweep voltammetry (LSV) polarization curves (iR corrected) in 1 M KOH. **b** *η* required to reach − 10 to − 100 mA cm^−2^. **c** Corresponding Tafel plots. **d** HER LSV polarization curves (iR-corrected). **e** *η* required to reach − 10 and − 100 mA cm^−2^. **f** Corresponding Tafel plots. **g** Chronopotentiometric stability test for NiCo-SAD-NC and NiCo-NP-NC in 0.5 M H_2_SO_4_ and 1 M KOH at *j* of − 100 mA cm^−2^. The insets reveal that faradic efficiency of NiCo-SAD-NC for HER at -100 mA cm^−2^ in 0.5 M H_2_SO_4_ (right) and 1 M KOH (left). Reproduced with permission from Ref. [61]. Copyright 2021 Springer Nature

The HER is crucial for producing gaseous hydrogen through a series of multistep reactions occurring at the electrode surface. The Volmer reaction, or discharge reaction, represents the initial step of HER. This involves the transfer of an electron to the electrode, followed by the coupling with a proton to facilitate adsorption at an unoccupied active site on the electrode, resulting in the formation of an adsorbed hydrogen atom [201]. Hydronium ions (H_3_O^+^) and H_2_O molecules are the proton source in acidic and alkaline electrolytes, respectively [201]. Moreover, hydrogen gas can be produced via two different reaction pathways [201]. The first pathway, known as the Heyrovský reaction, involves the transfer of a second electron to an adsorbed hydrogen atom, which coincides with the transfer of another proton from the solution to form hydrogen gas [201]. The second pathway involves the recombination of two adsorbed hydrogen atoms on the electrode's surface toward hydrogen gas through the Tafel reaction [202, 203], confirmed for Pt to produce hydrogen. Usually, through the calculation of the Tafel slope from the HER polarization curve, it is possible to evaluate the kinetics of the reaction and the rate-determining step [204]. HER operates via an adsorbed hydrogen intermediate (H*). The Gibbs free energy for H* adsorption on the catalyst surface (ΔG_H∗_) is a critical parameter for evaluating both the adsorption of H* and its subsequent desorption for hydrogen gas formation. Such measurements can be effectively utilized within a HER free energy diagram to analyze these interactions on metal surfaces and derive crucial mechanistic information. Quantum chemical calculations confirm the feasibility and capability of ΔG_H∗_ to describe catalysts for HER [204]. Catalysts are applied to reduce the energy barrier and stimulate the reaction rate. To reduce the kinetic barrier for water splitting reaction, bimetallic catalysts including noble and non-noble metals and metal oxides have been widely applied to enhance the performance [205–207]. Noble metal-based catalysts are significantly more effective compared to other metal catalysts for water splitting via reducing the energy barrier [208]. Single-metal atoms offer unique opportunities to constitute every metal atom active and readily available for the interaction with the reactants and their transformation to the desired products [61]. In addition, bimSACs can introduce new tools to modulate the local electronic structure of the catalytic active centers, as well as to bring added values through the development of atomic-level synergistic effects, improving HER kinetics [61]. For example, first-principles calculations indicated that the synergistic interactions developed in a NiCo bimSAC interface elevated the d-band center facilitating instant water dissociation and improved proton adsorption, accelerating HER kinetics in alkaline and acidic conditions [61]. Inspired by these theoretical predictions, Kumar et al. [61] developed a NiCo bimSAC structure on (N–C), which displayed unique pH-universal HER activity, with the requisite of only 54.7 and 61 mV *η* at − 10 mA cm^−2^ for acidic and alkaline media, respectively. A study on indirect catalytic synergies in C-supported bimSACs reported that spatially and electronically isolated Ru and Pt atoms, despite their isolated nature, the Ru-Pt bimSACs revealed up to 15-fold improved activity for HER compared with their monometallic counterparts [83]. In another example, a NiCo bimSAC showed extraordinary pH-universal catalytic activity toward HER [61]. The NiCo bimSAC delivered remarkable HER activity in alkaline media with *η* of 61 and 189 mV reaching − 10 and − 100 mA cm^−2^, which was superior to individual Ni and Co SAs, as well as Pt SAs (Fig. 11a, b) [61]. The Tafel slope (55 mV dec^−1^) of the NiCo bimSAC (Fig. 11c) was equivalent to that of 20% Pt-C (55.6 mV dec^−1^) [61], revealing that the HER followed the Volmer–Heyrovsky mechanism where electrochemical desorption was the rate-determining step [124]. The NiCo-bimSAC also revealed exceptional HER performance under acidic media (0.5 M H_2_SO_4_) (Fig. 11d, e) [61]. The high activity of the NiCo bimSAC, similar to that of Pt-C, is defined by the rate-determining step, where two hydrogen atom intermediates desorb and form molecular hydrogen (Tafel step) [103] (Fig. 11f) [61]. The superior catalytic activity of the NiCo-SAD-NC under acidic and alkaline media was further verified by the small charge transfer resistance (*R*_CT_), combined with a high electrochemically active surface area (ECSA) of 13.05 cm^2^. Interestingly, the nanoparticulate analogue catalyst of NiCo-NP-NC displayed an ECSA only 6.8 cm^2^ [61], highlighting the fast electron transfer kinetics with more active sites in the case of the bimSAC, responsible for the enhancement of the performance for HER [61]. The stability performance of the NiCo bimSAC showed exceptional performance under acidic and alkaline media without noticeable degradation (Fig. 11g) [61].

Zhao et al. introduced a novel MXene surface modification strategy by pre-adsorbing L-tryptophan molecules, which facilitated the attachment of Co/Ni dual atoms on the surface of Ti_3_C_2_T_x_ through the formation of N–Co/Ni–O bonds [202]. This modification strategy leverages electron delocalization arising from the terminated O atoms on the MXene support and from the N atoms in the L-tryptophan anchoring groups. The Co and Ni dual atoms provided optimal adsorption strength of intermediates [65], leading to a synergistic interaction which significantly enhanced the intrinsic activity toward the HER. With this strategy *η* of only 31 mV at *j* of 10 mA cm⁻^2^ was achieved [209]. Hetero-bimSACs developed on a nano-support of H_x_MoO_3_ (PtM/H_x_MoO_3_ catalysts, where M = Ag, Au, Pd, Rh) and with a low noble metal loading (wt% < 1%) exhibited outstanding efficiency for HER [210]. Experimental data showed substantial improvements in electrochemical surface area and HER kinetics, particularly for PtPd/H_x_MoO_3_, which demonstrated low *η* (10 mV at 10 mA cm^−2^) and a Tafel slope of 36 mV dec^−1^. Mechanistically, the catalysts enhanced water dissociation by upshifting the d-band center toward the Fermi level, thus increasing the adsorption strength of water molecules and accelerating the splitting and the reaction rate. The Pt incorporation reduced hydrogen evolution and stabilized the catalytic performance over prolonged periods, outperforming commercial Pt/C and monometallic catalysts. Dual-metal sites in a Co-catecholate (Co-CAT) framework crystalized in nanorod shape over a carbon cloth support were engineered through the doping of Ru, Ir, or Rh for efficient OWS [211]. The nanorods were approximately 1.0 µm in length and 85.0 nm in diameter. Among these, RuCo-CAT exhibited exceptional bifunctional catalytic activities, also surpassing the performance of the benchmarked Pt/C catalysts [211]. XPS and EXAFS analyses revealed strong electronic interactions between Ru and Co atoms, facilitating charge transfer and improving catalytic activity. The band structure and DOS calculations indicated that Ru doping enhanced the electrical conductivity and charge transport properties of the catalyst. The Ru sites in RuCo-CAT exhibited an adsorption energy of hydrogen (ΔG_H_*) close to zero, optimizing the binding and release strength for hydrogen intermediates. The Ru sites also significantly reduced the energy barrier for water dissociation, enhancing the HER kinetics. The Co sites in RuCo-CAT showed a lower Gibbs free energy for the rate-determining step (formation of *OOH intermediate) compared to undoped Co-CAT. This reduction in energy barriers leads to improved OER activity. This synergy between the catalytic sites improved the electronic properties leading to superior bifunctional performance for both the OER and HER achieving an *η* of 38 mV at *j* of 10 mA cm^−2^, outperforming commercial Pt/C catalysts. The same catalyst required an *η* of 200 mV for OER at 10 mA cm^−2^, surpassing the activity of RuO_2_ benchmark [211]. Ge et al. [212] introduced an innovative catalyst design based on the concept of electronegativity difference toward the regulation of bimSACs electronic states. They fabricated a highly efficient HER catalyst using Ru and Ni-modified MoS_2_ (Ru/Ni-MoS_2_). Studies revealed that the Ru SAs were strongly bonded to the Ni SAs due to their significant electronegativity difference. Most Ru atoms were coordinated with S atoms and distributed atop the Ni sites and occasionally on other favorable sites. In the Ru/Ni-MoS_2_ nanosheets, Ni atoms substituted Mo atoms in the MoS_2_ lattice, providing a uniform distribution of active sites. DFT calculations revealed that the introduction of Ni atoms into MoS_2_ created an electron-rich environment, enhancing the adsorption of hydrogen on S atoms bonded to Ni (S–Ni) and hydroxyl groups on Ru atoms. The synergistic function of Ru and Ni effectively lowered the energy barrier for the water dissociation step and enhanced the HER performance [212]. Moreover, Guo et al. [213] proposed a two-step pyrolysis method to develop Zn–Co bimSAC for effective HER. The catalyst consisted of Zn and Co atoms asymmetrically coordinated with S and N atoms within a carbon matrix (SNC), as confirmed by HAADF-STEM showing a high density of bright dots representing the atomic dispersion of Zn and Co atoms. The Zn_1_Co_1_–SNC catalyst demonstrated *η* of 49 mV at *j* of 10 mA cm^−2^, significantly lower than commercial Pt/C and Zn/SNC catalysts. It also exhibited a Tafel slope of 48 mV dec^−1^, indicating fast reaction kinetics. Electrochemical impedance spectroscopy showed that Zn_1_Co_1_–SNC had a lower charge transfer resistance compared to Zn/SNC, indicating improved charge transfer due to the presence of the dual-metal SA sites. This structure facilitated a redistribution of electrons across the active sites, enhancing the conductivity and charge transfer properties of the catalyst. The synthesized Zn_1_Co_1_-SNC catalyst exhibited strong interaction between Zn and Co, leading to the hybridization of their *d*-orbitals, and to a more delocalized electronic structure. This hybridization enhanced the DOS near the Fermi level, facilitating better electron mobility and charge transport [69]. The exploration of bimSACs in electrocatalytic applications, particularly water splitting, has not been extensively pursued to date but represents a rapidly emerging area with significant potential. These catalysts offer exciting opportunities for breakthroughs in catalytic technology due to their unique synergistic interactions at the atomic level, which could greatly enhance catalytic efficiency and selectivity. The field holds considerable promise for advancing catalytic research and potentially revolutionizing energy conversion technologies in the near future.

### Oxygen Evolution Reaction

**Fig. 12:**
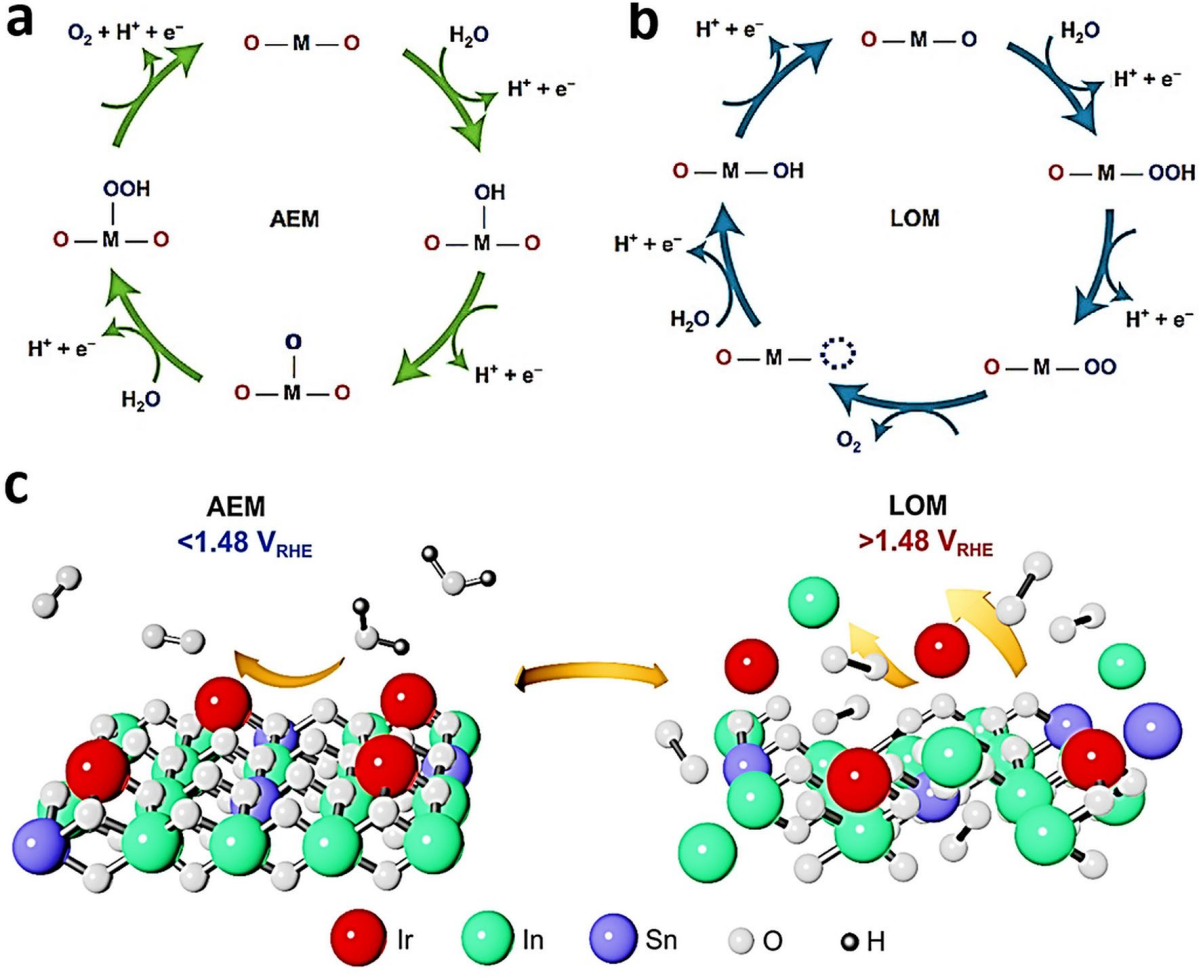
**a** Conventional AEM. **b** LOM (M represents the active sites). Reproduced with permission from Ref. [219]. Copyright 2023 John Wiley and Sons. **c** Stability assessment of Ir single-atom OER electrocatalysts. Reproduced with permission from Ref. [224]. Copyright 2023 Elsevier

In the water splitting reaction, OER is recognized as a primary bottleneck [214] due to its slow kinetics [215], which significantly impedes the efficiency of energy conversion [216, 217]. The inherently sluggish kinetics of the multistep proton (H^+^) coupled with electron (e^−^) transfer processes [218], necessitate a high *η*, leading to low energy efficiency [219]. That is why OER is less explored as compared to HER in water splitting [220]. OER involves four coupled electron/proton transfer steps in both acidic and alkaline media [217]. OER is a pH sensitive reaction. Under acidic conditions, water molecules (H_2_O) are oxidized, whereby H^+^  + e^−^ pairs and oxygen molecules (O_2_) get released [221]. On the other hand, for alkaline environments, hydroxyl groups (OH^−^) are oxidized to H_2_O and O_2_ with simultaneous release of electrons [222, 223]. Xie et al. [219] proposed an OER pathway under alkaline media based on adsorbate evolution mechanism (AEM) (Fig. 12a) and on lattice oxygen evolution mechanism (LOM) (Fig. 12b). In the primary step, a hydroxyl anion (OH^−^) from the electrolyte adsorbs on the active site (M) to create M-*OH species (where “*” represents the adsorption of the O-intermediate on M) by one-electron oxidation. Paired H^+^ and e^−^ extraction from M-*OH results in M-*O [219]. In the AEM mechanism pathway, the M-*O combines with an OH^−^ to form M-*OOH. After the reaction with one more OH^−^, O_2_ and H_2_O are produced. In LOM pathway (Fig. 12b), lattice O_2_ in the oxide catalyst can be immediately involved in the O–O pairing and contribute in the OER, avoiding the limitations of the adsorption energy scaling relationship of the AEM [219].

Considerable research efforts have been focused on improving the activity or selectivity of SACs toward OER. The stability is another important feature of a successful catalyst. Zlatar et al. [224] employed online inductively coupled plasma mass spectrometry (ICP-MS) to evaluate the stability of Ir SAC with highly dispersed Ru active sites and compared them to commercial benchmark nanoparticulate catalysts like IrO_2_ and RuO_2_. The SACs were prepared via surface organometallic chemistry and supported on indium-doped tin oxide (ITO). It was found that SACs, while active, tend to show lower stability compared to traditional catalysts, which illustrates an inverse activity–stability relationship [224]. This instability may stem from a shift in the O_2_ evolution mechanism from adsorbate evolution to lattice O_2_ involvement, facilitated by the catalyst's interaction with the support. In traditional catalysts, the OER typically occurs via adsorbate evolution, where water molecules are adsorbed on the catalyst surface and dissociate to release oxygen (AEM) [224]. The study suggested that in SACs, the mechanism shifted toward LOM, where the O_2_ involved in the reaction may partially come from the oxide lattice of the catalyst support itself, not just from the water molecules. This shift can lead to the degradation of the catalyst support, contributing to the overall instability of the SACs (Fig. 12c) [224]. This was validated by the rise in Tafel slope, corresponding to the dissolution onset of the ITO support [224]. Future research might focus on designing catalyst supports that can enhance stability without limiting the catalytic activity of SACs or exploring alternative materials that resist the destabilizing effect of high activity levels during OER.

The mechanism depicted by Xie et al. [219] offered an improved overview for the possible OER involved mechanisms, presenting key differences in the number of e^−^ and H^+^ transfer in specific steps. Electrocatalysts are considered as promising candidates to enable the required electron transfer steps, as well as to facilitate the emergence and breaking of chemical bonds [41, 225]. Therefore, the advancement of highly dynamic and stable OER catalysts is crucial. Precious metal catalysts have been intensively explored as benchmarks in acidic environments due to their high stability and activity. However, their natural scarcity and high costs hamper their broader application and applicability in commercially competitive electrolyzers [103]. Therefore, to fully exploit the application potential of electrolyzers, the OER catalyst materials must be cost-effective with appropriate OER kinetics and durability [219]. Continuous efforts have been focused toward the development of effective electrocatalysts for OER in alkaline conditions [219]. TM oxides/hydroxides/oxyhydroxides have been explored as potent OER catalysts in alkaline water electrolysis, while SACs and bimSACs are emerging as a novel class of catalysts with well-defined active sites that present an encouraging solution to address the existing challenges [175, 226–228]. Replacing precious metal atoms and critical elements with cost-effective earth-abundant alternatives, while retaining or surpassing the activity of the noble metal sites. Moreover, SACs and bimSACs offer the potential to modulate the electronic structure of the active sites via the geometric coordination environment, the interactions with the support, and with the adjacent metal atoms [227]. The selection of the host support and tuning of the local atomic structure make it possible to engineer a unique SAC offering improved catalytic features [227]. Engineering of bimSACs with high metal loadings also holds great promise in energy conversion and storage applications [229]. In this regard, such a strategy was applied for the development of bimSACs based on Ni and Fe for OER [229]. In this catalyst, the Ni and Fe atoms co-existed on a graphene support and proved to undergo strong synergistic effects, which significantly promoted the charge transfer and reversible redox cycles with a small *η* of 247 mV at 10 mA cm^−2^ current density in KOH electrolyte [230]. Bai et al. synthesized Co-, Fe-, and Ni-bimSACs from their SA precursors via in situ electrochemical transformation. The metal SAs acquired a unique molecular-like structure, which enhanced the catalytic efficiency for OER [230]. All stated catalysts showed metal–metal cooperation contributing to the crucial step of O–O bond formation, which is the most energy-intensive step of the reaction. The oxidation state of each metal in the bimetallic site affected the flow of electrons during the reaction. Fe(III)/Fe(IV) oxidation states changed reversibly to facilitate the transfer of electrons needed for O–O bond formation [230]. In the Fe-Co bimSAC, Fe reduced the redox potential of Co(III)–OH to Co(IV) = O, which enhanced the catalytic activity achieving lower *η*. This catalyst displayed kinetic differences related to the Lewis acidity of the metals, which affects proton transfer. While the catalysts generally follow a shared mechanistic framework where Co(IV) = O as the active intermediate, the second metal ion modulates this activity by altering the redox potential and sequence of electron or hydroxide ion transfers [230]. Unlike traditional molecular catalysts, these double-atom catalysts, embedded in an N-doped carbon matrix, provided improved stability and avoided the degradation commonly observed in homogeneous complexes with discrete organic ligands. This study emphasized the significant catalytic efficiencies of these double-atom catalysts, while providing a novel platform for the fundamental understanding of OER mechanisms. Such catalysts represent a promising avenue for future research in electrocatalysis, potentially impacting the development of efficient and sustainable energy technologies.

**Fig. 13:**
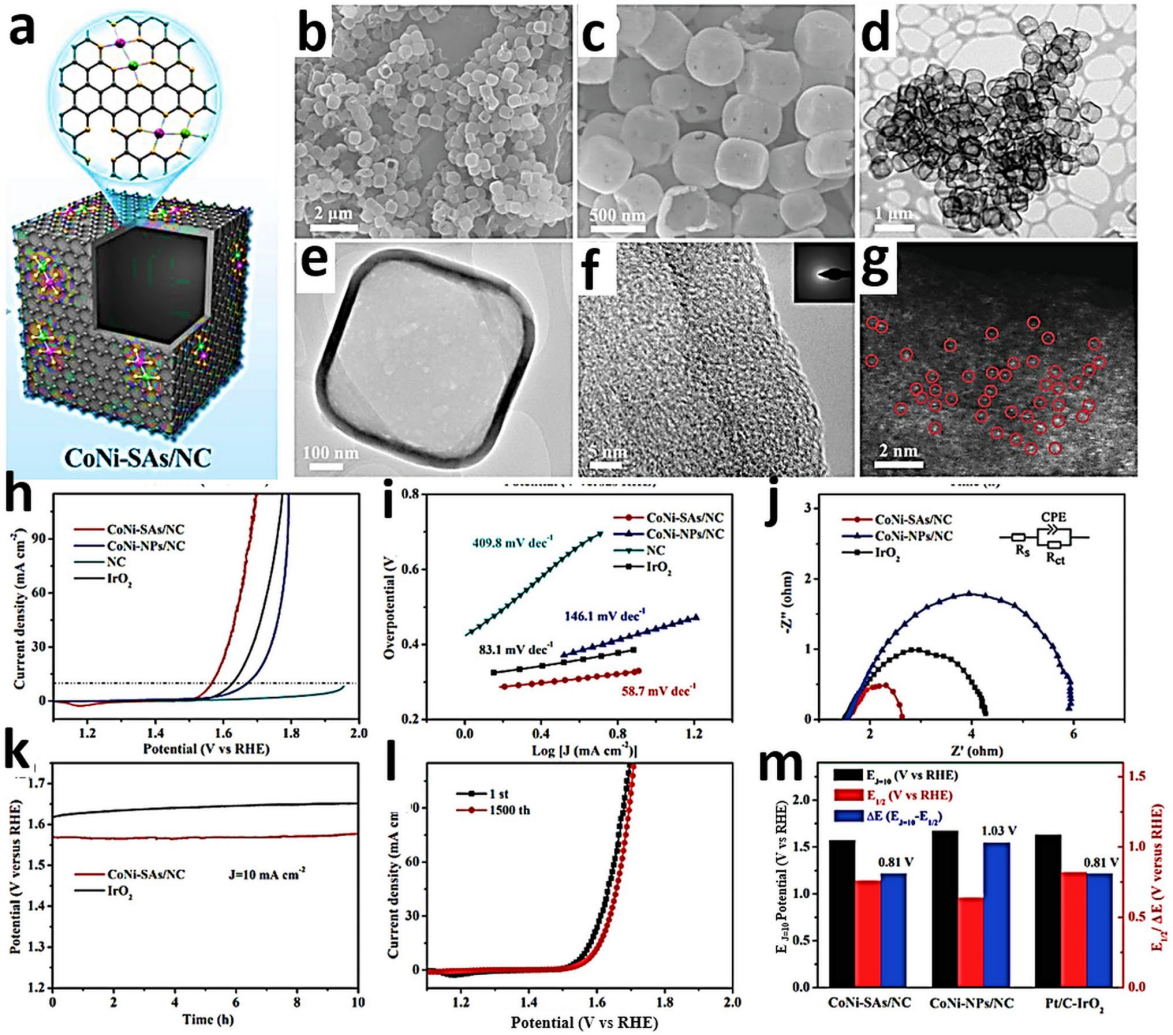
**a** Schematic of CoNi-SAs/NC. **b, c** SEM, **d, e** TEM, **f** HRTEM and, **g** HAADF-STEM of CoNi-SAs/NC. **h** OER polarization curves. **i** Tafel plots, and **j** electrochemical impedance spectroscopy plots at 1.62 V. **k** Chronopotentiometry response of CoNi-SAs/NC at a constant *j* of 10 mA cm^−2^ in contrast with that of IrO_2_. **l** Polarization curves of CoNi-SAs/NC before and after 1500 cycles. **m** OER potential at *j* of 10 mA cm^−2^ (E_J = 10_), ORR half-wave potential (E_1/2_), and their difference (ΔE) of prepared catalysts. Reproduced with permission from Ref. [59]. Copyright 2023 John Wiley and Sons

The rational design of SACs with a homogeneous structure and adaptable active sites for OER requiring a 4e^−^ reduction mechanism remains a formidable task [65, 231]. Nevertheless, substantial advances have been witnessed with the use of bimSACs. Tang et al. successfully developed a diatomic Fe-Co catalyst where the SAs are coordinated to N and O atoms, respectively, through bridging N and O atoms [232]. This Janus-like quaternary dimer (FeCo-N_3_O_3_@C) demonstrated significant stability and reactivity toward both the OER and ORR [232]. Experimental and theoretical studies indicated that the strong coupling effect between the Fe–N_3_ and Co–O_3_ moieties enhanced the bifunctional performance [232]. It also modified the d-orbital energy levels of the metal atoms optimizing the adsorption and desorption of oxygenated intermediates and improving the reaction kinetics for OER and ORR [232]. Lu et al. [233] employed a doping strategy to create abundant dual-atom sites within single-phase oxide catalysts, in particular RuO_2_. This system is not an actual bimSAC since the RuO_2_ is a continuous phase; however, this catalyst featured a dual-atom active site structure with Mn and Ru atoms, leveraging the electronic interactions and the development of bonds between Ru/Mn to optimize catalytic activity. XPS and DOS analysis showed that the oxidation state of Ru sites decreased and the electron density of the Mn sites was enriched [233]. The catalyst demonstrated high ORR and OER activities, with electrochemical tests revealing that Mn-RuO_2_ had a lower *η* for both ORR and OER compared to undoped RuO_2_ and MnO_x_. Specifically, the Mn sites facilitated ORR by optimizing the adsorption energy of O_2_ species, while Ru sites were responsible for the OER process by reducing the adsorption capacity for O_2_ intermediates, thus lowering the energy barrier for O_2_ desorption. The Mn^3+^ ions, due to their enriched electron density compared to Ru^4+^, served as electron donors, which is crucial for facilitating the ORR process. The Mn sites, enriched with electrons, helped in the dissociation of O_2_ molecules, making it easier for O_2_ to adsorb and reduce on the catalyst surface. The dual-atom sites enhanced the co-adsorption of O_2_*/OOH*, significantly boosting ORR activity. This co-adsorption lowered the energy barriers for the intermediate steps in the ORR, particularly the desorption of OH*, which is often the rate-limiting step in the ORR. The catalyst's charge redistribution and electronic structure modulation confirmed through XPS and DOS analysis, further supported these findings by showing a favorable shift in the d-band center away from the Fermi level [71]. In another example, a Fe–Cu hetero-bimSAC with coordination motifs of Fe–N_6_ and Cu-N_1_S_2_ type was reported. The FeCu-DSAs/NSC catalyst, benefiting from the synergistic effect of dual-atom sites and asymmetric heteroatom coordination, exhibited significantly enhanced catalytic performance [234]. This improvement was evidenced by a smaller potential gap between E_η10_ and E_1/2_, ΔE = 0.647 V, compared to benchmark counterparts (Pt/C + IrO_2_ and Cu-ISAs/NSC) [234]. A catalyst (reported as NiFe LDH A-FeSACoSA-FeCoAlloy-CNT/NC) was developed by integrating a nickel–iron layered double hydroxide (NiFe LDH) coating over an iron-cobalt dual single-atom catalyst (FeSACoSA) and iron-cobalt nanoalloy (FeCoAlloy), which was embedded within carbon nanotubes (CNTs) and a nitrogen-doped porous carbon framework (NC). The catalyst demonstrated exceptional electrocatalytic performance with multifunctional activity toward both OER and ORR [235]. The Fe SAs were identified having an Fe–N_x_ type coordination environment, confirmed by XANES spectra and resembling iron phthalocyanine. EXAFS showed both cobalt Co–N bonds as well as Co–Co bonds, confirming the coexistence of cobalt SAs and metallic type cobalt in the FeCo alloy, while the Ni K-edge EXAFS revealed Ni–O and Ni–Ni bonds in the NiFe LDH structure. The NiFe LDH enhanced OER efficiency because the d-orbital electrons in the LDH could interact strongly with the reaction intermediates, facilitating the adsorption and desorption of these intermediates during the process. This leads to a lower activation energy for the reaction, thereby enhancing the OER efficiency [235]. The FeSACoSA and the FeCo alloy synergistically provided the active sites for highly efficient ORR. The Fe and Co SAs provided optimal adsorption energies for O_2_ intermediates (O_2_, OOH, O, OH). The strong adsorption for the initial O_2_ molecule lowered the energy required for bond cleavage, while the nanoalloy sites were rich in electrons and facilitated the subsequent reduction steps, ensuring a smooth and efficient reaction pathway [235]. A boron (B)-coordinated bimSAC structure was also reported with iron and nickel dual sites, where both metal atoms were coordinated with four N and one B atom (FeN_4_B-NiN_4_B). The incorporation of B into the first coordination sphere of FeN_4_ and NiN_4_ atomic sites altered their geometry and electronic structure by creating “Fe–B–N” and “Ni–B–N” bridges. The B doping enhanced the electronic conductivity and induced asymmetric charge distribution around the Fe and Ni sites, which was beneficial for the stronger adsorption of reactants and desorption of O_2_ intermediate products. This modification resulted in significantly improved catalytic performance compared to their metal-only counterparts. The FeN_4_B sites demonstrated superior ORR activity with a half-wave potential of 0.9 V vs RHE, attributed to weakening of the Fe–O binding energy facilitating the release of reduced O_2_ species. The introduction of B also resulted to a significantly reduced *η* (0.224 eV) for the reaction determining step involving the conversion of OOH* to OH*. The NiN_4_B sites, on the other hand, were primarily responsible for the enhanced OER activity, showing a lower *η* of 1.618 V at 10 mA cm^−2^, comparable to commercial RuO_2_ catalysts. B doping enhanced the interaction between the Ni center and O_2_ intermediates, helping with the deprotonation of the OH* species [235]. Hetero-bimSACs with noble metals and 3*d* TMs were also developed for OER (e.g., NiPd, CuPd, CuPt) embedded in N–C [236]. The catalyst exhibited unique properties such as strong adsorption of oxygenated intermediates, crucial for OER. This strong adsorption, however, must be balanced to avoid excessive binding that could hinder reaction efficiency. The catalysts showed varying adsorption energies for intermediates like *OH, *O, and *OOH, with the dual-metal sites providing a more favorable environment compared to SACs analogues. The adsorption of OH took place on the metal dimer sites with the subsequent adsorption of another OH and formation of *OH-*OH. The interaction between the dual-metal atoms (e.g., Ni and Pd) facilitated the stabilization of this intermediate, which was then transformed into *O after deprotonation. The key finding was the ability to facilitate direct *O–*O coupling, bypassing the formation of *OOH intermediates, which is a limiting step in conventional single-site mechanisms. This direct coupling reduced the overall energy barrier for OER. The NiPd@NC catalyst demonstrated significantly lower *η* (0.06 V at 10 mA cm^−2^) compared to traditional catalysts (like IrO_2_, RuO_2_, and NiFe oxides) which typically demonstrate *η* in the range of 0.25 to 0.35 V at 10 mA cm^−2^ [236]. A Co–Ni bimSAC was reported as bifunctional electrode material, synthesized by embedding in N-doped hollow carbon nanocubes (CoNi-SAs/NC) (Fig. 13a) [59]. The atomically isolated bimetallic configuration in CoNi-SAs/NC was confirmed by using microscopic techniques (SEM, TEM, HRTEM, HAADF-STEM, Fig. 13b–g) [59]. The CoNi-SAs/NC hybrid was studied as OER electrocatalyst (Fig. 13h–m) in alkaline medium [59]. It displayed an unusual bifunctional catalytic performance both for ORR and OER. This resulted in improved performance in rechargeable zinc–air batteries with very high energy conversion efficiency, low *η*, and high reversibility. These qualities made it superior to other similar systems and even state-of-the-art precious metal catalysts [59].

**Fig. 14:**
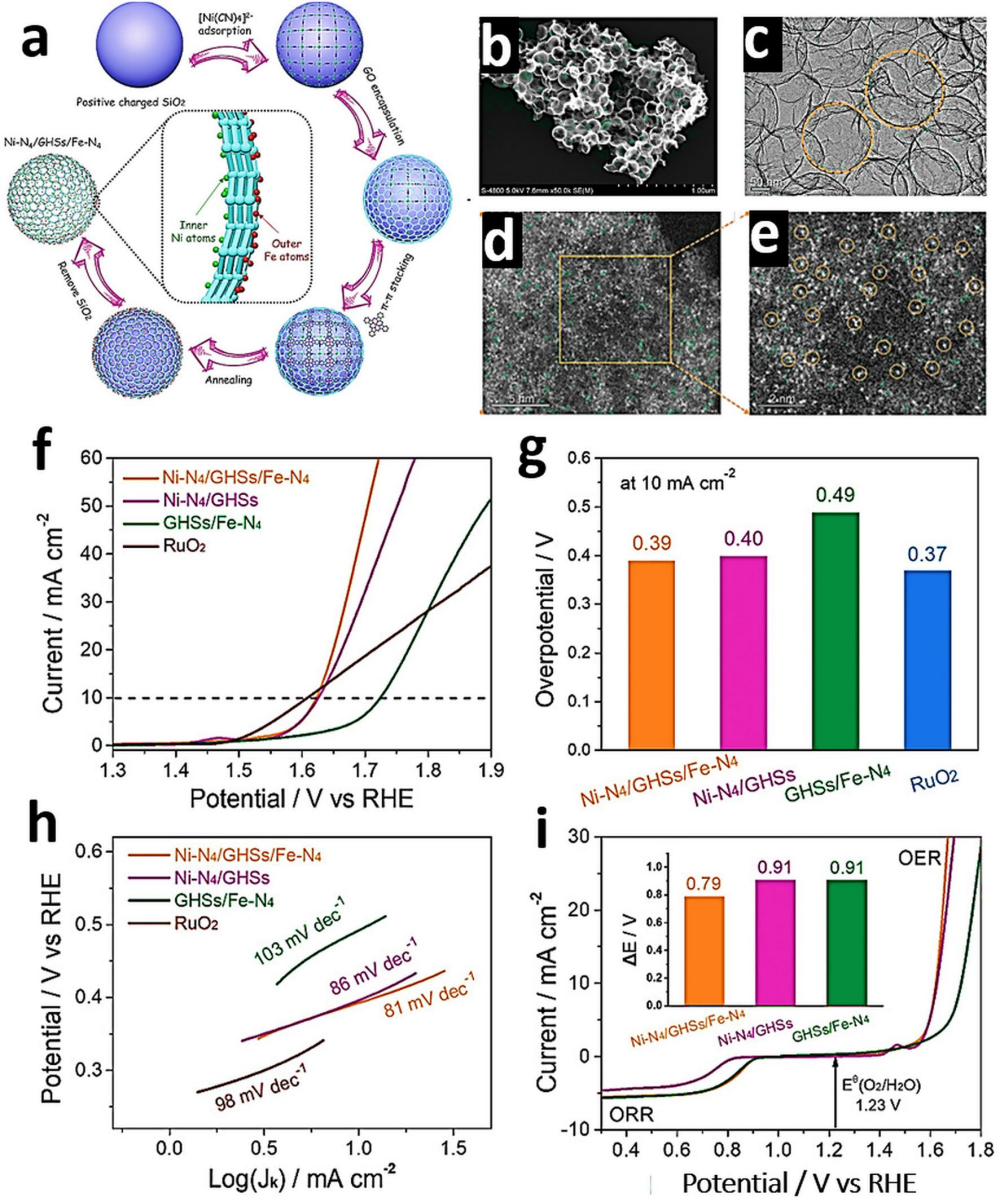
**a** Schematic of the Ni-N_4_/GHSs/Fe-N_4_ catalyst synthesis. **b** SEM, **c** TEM, **d, e** aberration-corrected STEM images of Ni-N_4_/GHSs/Fe-N_4_. **f** OER polarization curves in O_2_-saturated 0.1 M KOH. **g** Bar plots of *η* obtained at 10 mA cm^−2^. **h** Tafel plots reordered from, **i** overall polarization curves of catalysts. Reproduced with permission from Ref. [238]. Copyright 2024 John Wiley and Sons

SAs coordinated with nitrogen in carbon supports have stimulated widespread attention and emerged as a highly promising field and active research frontier in a comprehensive range of key reactions related to renewable energy exploitation [29, 117, 160, 237]. In an interesting approach, a bifunctional electrocatalyst using a Janus structure incorporated dual single atomic sites (Ni-N_4_ and Fe-N_4_) on the opposite sides of hollow graphene spheres (GHSs) (Fig. 14a) [238]. This structure enhanced the catalyst's activity for both the OER and the ORR, which are critical for the efficiency of rechargeable metal–air batteries. The morphology and structure of Ni-N_4_/GHSs/Fe-N_4_ hybrid were confirmed with SEM, TEM, and STEM techniques (Fig. 14b–e) [238]. The Ni or Fe SAs were confirmed to be coordinated with four N atoms via the formation of a Ni–N_4_ or Fe-N_4_ planar arrangements. The Janus configuration of the Ni–N_4_/GHSs/Fe–N_4_ catalyst leverages the distinct catalytic properties of the Ni and Fe sites, strategically separated by the graphene layer. This separation minimized cross-reaction interference, allowing each type of site to specialize in either ORR or OER. Such a setup enhanced the overall efficiency and effectiveness of the catalyst leading to an admirable bifunctional electrocatalytic performance, in which the outer Fe-N_4_ sites dominantly contributed toward the ORR, while the inner Ni-N_4_ clusters were accountable for exceptional activity toward the OER, as shown in Fig. 14f–I [238]. The Fe–N_4_ sites are particularly effective for ORR due to their ability to facilitate the 4e^−^ transfer mechanism, which directly reduced O_2_ to H_2_O without forming peroxide intermediates. This efficiency was ascribed to the electronic structure of Fe–N_4_, where the Fe center can easily cycle between different oxidation states, effectively transferring electrons to the O_2_ molecules. The Ni–N_4_ sites provided the necessary electronic structure to facilitate the deprotonation of water molecules and the subsequent formation of O_2_. The high oxidation state of Ni in Ni–N_4_ was crucial for attracting electrons from OH- ions, facilitating the formation and release of O_2_. In OER, Ni–N_4_ sites underwent a series of oxidation state changes improving the absorption and release of electrons, which were crucial for breaking the O–H bonds in water and forming O=O bonds in molecular O_2_ [238].

**Fig. 15:**
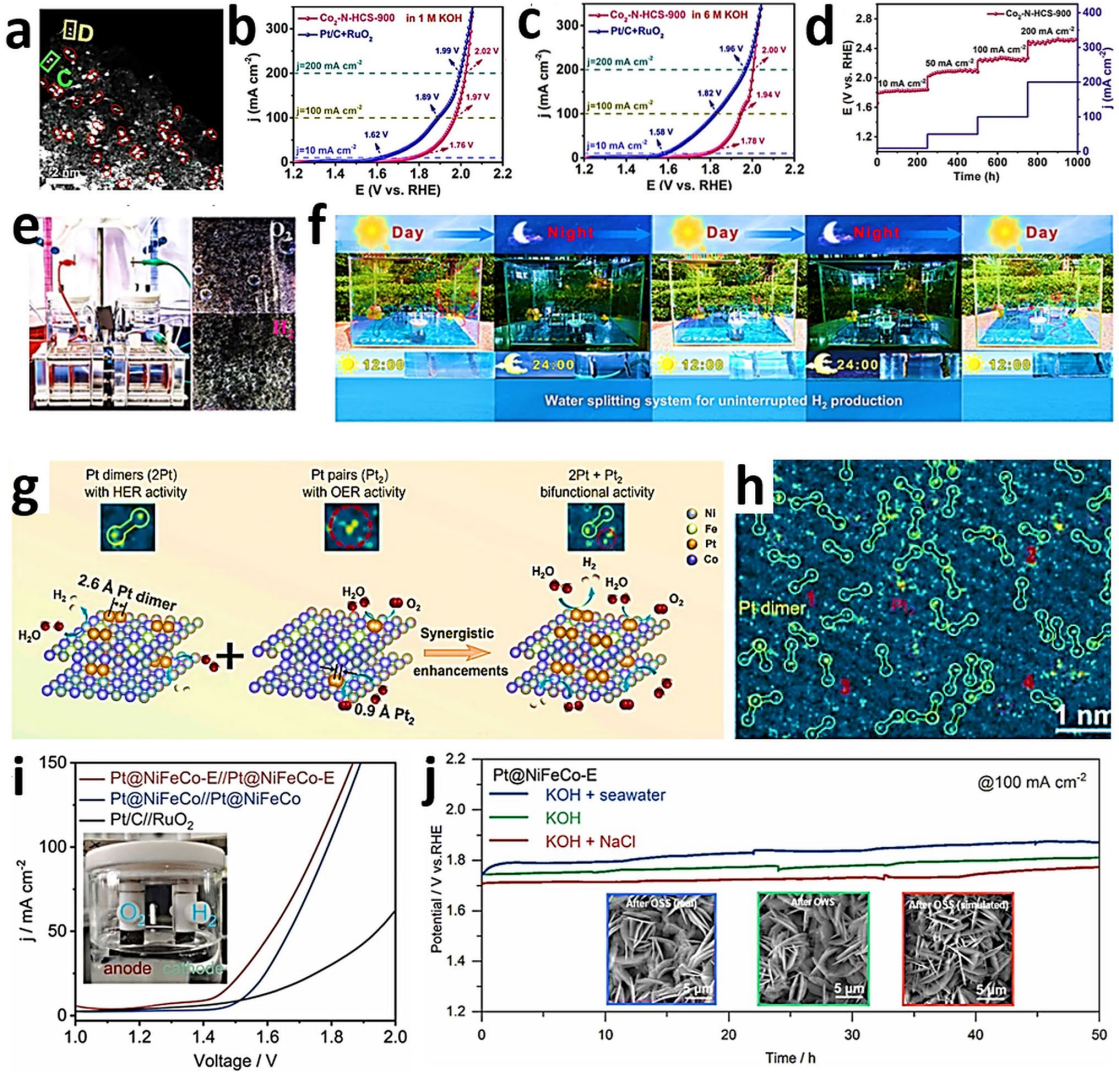
**a** Aberration-corrected HAADF-STEM image of Co dual atoms marked by red circles, LSV curves recorded for **b** ORR, **c** OER, **d** HER in 1 M KOH. **e** Digital image of the water splitting. Reproduced with permission from Ref. [86]. Copyright 2023 Springer Nature. **f** Photograph of series water splitting system driven by Co_2_-N-HCS-900 during day and night over 48 h. **g** Schematic of Pt dimers and Pt pairs on NiFeCo-E and their bifunctionality toward water splitting catalysis. **h** Atomic-resolution HAADF-STEM image of Pt@NiFeCo-E. **i** OWS performance of the cells with Pt@NiFeCo//Pt@NiFeCo and Pt@NiFeCo-E//Pt@NiFeCo-E. **j** Chronopotentiometric curves of OWS in alkalized freshwater, simulated seawater, and real seawater for Pt@NiFeCo-E//Pt@NiFeCo-E at 100 mA cm^−2^ for 50 h; the insets are the morphologies after tests. Reproduced with permission from Ref. [248]. Copyright 2024 Elsevier

Electrochemical water splitting has emerged as one of the most promising methods for producing green hydrogen [239], which can significantly promote the goals set by international organizations toward C neutrality and energy security [240]. Substantial efforts by the research community have been dedicated to developing high-performance electrocatalysts for the two half-reactions involved in water electrolysis [241]. In the OWS process, the OER is a bottleneck requiring a high *η* to achieve the desired *j* compared to the HER process [242, 243]. The low electrochemical efficiency in OWS electrolysis is also attributed to the slow kinetics of both the HER and OER [241, 244, 245], since both HER and OER demand higher *η* to overcome the inherent reaction energy barrier [244]. Therefore, developing efficient electrocatalysts with improved electronic conductivity, large active surface area, and strong catalytic activity for water splitting is critically important and represents a significant area for innovation [246]. BimSACs offer more opportunities to enhance the kinetics and multifunctional performance for the OER and HER. However, the rational design of efficient multifunctional bimSACs remains challenging. Recently, Wang et al. achieved controllable synthesis from Co NPs to CoN_4_ SACs and further to Co_2_N_5_ bimSACs (Fig. 15a) [86]. Interestingly, their reported strategy extended to the fabrication of 22 distinct bimSACs. Notably, spin-state-tailored Co_2_N_5_ bimSACs achieved an ideally balanced adsorption/desorption of intermediates, resulting in superior multifunctional activity (Fig. 15b–d). A water electrolysis device was established using only one catalyst of Co_2_-N-HCS-900 (Fig. 15e) [86]. Furthermore, these catalysts enabled water splitting systems to operate continuously for 1000 h and supported solar-powered water splitting systems for uninterrupted large-scale hydrogen production throughout the day and night (Fig. 15f) [86]. BimSACs are inspiring the development of emerging electrocatalysts by leveraging synergistic interatomic interactions [84, 247]. However, the impact of these atomic interactions on catalytic selectivity and activity has yet to be clearly understood [248]. Zhang et al. prepared two types of Pt dual-atom active sites on cationic vacancy-rich nickel-based hydroxide (Pt@NiFeCo-E), where the Pt dimers (2Pt) attained an interatomic distance of 2.6 Å and Pt pairs (Pt_2_) a distance of 0.9 Å (Fig. 15g). The presence of evenly dispersed 2Pt dimers and Pt_2_ pairs was confirmed by HAADF-STEM (Fig. 15h). It was observed that Pt dimers were favorable for better HER performance, while Pt_2_ sites were active for OER. The coexistence of these two types of Pt dual atoms endowed bifunctional activity and enabled efficient OWS in alkaline media (Fig. 15i) at an *η* of 1.42 V, reaching *j* of 10 mA cm^−2^ and even sustaining 100 mA cm^−2^ for 50 h (Fig. 15j) [248]. In another example, the asymmetric structural evolution and dynamic hydrogen-bonding promotion mechanism of an atomically dispersed Co–Ni dual-metal site with an unprecedented N8V4 structure served as an efficient bifunctional electrocatalyst for OWS [249]. Confining dual atoms within the van der Waals gap of 2D-layered materials is anticipated to improve kinetic and energetic efficiency in catalytic processes. However, precisely assembling bimSACs within two adjacent 2D layers remains a significant challenge [250]. Jiang et al. [250] proposed an approach to assemble Ni and Fe bimSACs into the interlayer spaces of MoS_2_. By harnessing the exceptional benefits of diatomic species, this interlayer-confined structure exhibited enhanced adsorption strength of water molecules promoting bond cleavage on the confined metal active centers and demonstrated higher catalytic activity for acidic water splitting. Water electrolysis is usually connected with the use of high-purity water, which, if upscaled in a global setup, will exacerbate the shortage of freshwater resources [251]. However, seawater, which is abundant, offers a promising alternative technology for electrolysis, avoiding the consumption of freshwater resources, which is vital in geographical areas of high levels of drought [252]. Direct seawater electrolysis is viable, but it is hampered by the high salt concentrations poisoning the catalysts or blocking the pores and adsorption sites of the reactants. BimSACs with tailored local reaction environment of Co and Pt dual-atoms in a Ga-based liquid metal (referred to as Ga-CoPt) were reported as an effective catalyst for sea water OWS [252]. The synergistic effect between Co and Pt Combined with the high fluidity offered by the liquid Ga ascribing mobility to Co and Pt atoms facilitated the replenishment and reinforcement of active sites in the liquid Ga-CoPt. Such intricate structure substantially enhanced the catalytic activity and stability [252]. Operando Raman spectroscopy revealed the formation of key intermediate H_2_O adsorbed species on the Co atoms, where water was split generating protons and abundant H_3_O^+^ intermediates, forming and acidic environment around the active sites. The H_3_O^+^ and proton species were subsequently adsorbed on the Pt atoms and reduced to H* and subsequently to hydrogen. Thus, the synergy between Co and Pt atoms in liquid Ga was confirmed, with Co acting as the active site for water splitting, while Pt for the reduction of the H species [252]. Significant challenges remain for advancement in improving the kinetics toward OWS. Researchers are actively exploring novel materials and approaches aimed at advancing our understanding and capabilities in this critical area toward a broader societal and environmental goals, such as clean energy generation and C neutrality.

## Conclusions

Water splitting, a crucial process for hydrogen production, is at the forefront of renewable energy research. The search for efficient catalysts that can lower the energy barrier for this reaction has led to significant interest in SACs and bimSACs, also known as SA catalyst dimers. These catalysts, which feature pairs of metal atoms, exhibit unique properties that enhance their performance in water splitting, among other catalytic applications. They are designed to facilitate the electrochemical reactions involved in water splitting, which comprises two half-reactions (HER and OER). By leveraging the distinct electronic and catalytic properties of two different metal atoms, bimSACs can offer superior activity and stability compared to traditional catalysts. The metal pairs often create a synergistic effect, where, for example, one metal optimizes adsorption and activation of water molecules, while the other promotes the formation and release of reaction intermediates. Thus, they provide unique opportunities to modulate and optimize catalytic activity and physicochemical properties through strategic engineering.

In hetero-bimSACs, the water molecule typically interacts with both metal atoms. The distinct atomic environments provided by the two different metals can enhance the breaking of the O–H bond. One metal atom might act as an electron donor, weakening the bond, while the other can stabilize the formed hydroxyl group. Key methods toward achieving such precise functions include adjusting the coordination environment, increasing active site density, and leveraging the synergistic effects of adjacent atoms to influence activation and adsorption energies via charge redistribution. Different metal atoms can create a balance between adsorption energies of intermediates, avoiding the over-binding or under-binding that often limits the activity in monometallic catalysts. This balance is essential for the formation of key intermediates like *OH, OOH, and *O during the OER, and *H during the HER.

Several case studies have illustrated the superior performance of these catalysts in water splitting, fuel cells, and N_2_/CO_2_RR, often surpassing that of noble metals. Advances in theoretical models, machine learning, and experimental approaches are driving a deeper understanding of the structure-performance relationships, propelling these materials toward practical applications in sustainable energy technologies. Nonetheless, significant challenges persist, such as precise control over the coordination environment, over the distance between the metal atoms and thus of the metal binding sites, atomic-level characterization, and bottom-up or top-down synthetic strategies of catalysts with well-defined defect engineering or topochemical selective functional group installation as ligands for the single-metal atoms. Particularly for bimSACs, the distance control between the metal dimers is crucial since it critically influences the properties in a non-scalable way as a result of the quantum confinement effects. Moreover, interatomic distance substantially influences the metal–metal, metal–support, and metal–support–metal electronic interactions. In turn, these features are critical in the splitting of the water molecules. Adsorption of water via both oxygen and hydrogen on the catalyst can promote the homolytic or heterolytic splitting of the bond. The presence of two different active sites in hetero-bimSACs is crucial for furnishing on one side the two-electron hydrogen reduction and the 4e^−^ oxidation for the OER. Both of these steps require bond formations between H* and O* species, respectively. It is thus more efficient for this coupling to take place in a concerted way via two interacting M-adsorbate*||adsorbate*-M sites, rather than having a diffusion-controlled bond formation based on a single-site metal active center mechanism. The design and realization of structurally flexible bimSACs are also of high importance requiring advanced synthetic strategies. Structural flexibility facilitates the adoption of more effective geometries during the reaction transition states. Close proximity of dual active sites promotes formation of concerted intermediates, significantly lowering the energy barriers, while after bond activation and splitting, structural reorganization may promote interactions between newly formed intermediates and easier release of products.

BimSACs also exhibit unique properties not seen in NPs systems, such as rare valence states, reversible redox cycles, intense charge transfer phenomena, and controlled frontier orbital energy. The modulation of these interactions involving modifications in electronic structures and charge redistribution favorably alter the energy profiles of catalytic reactions. Therefore, bimSACs can excel in complex catalytic processes involving multi-step reactions or multiple reactants. With the aid of advanced in situ and operando characterization techniques, understanding of these catalysts under real reaction conditions can provide deeper insights, guiding future improved catalyst design and performance. Their versatility, compatibility with various supports, and adaptability to different reaction environments make SACs and bimSACs valuable for a wide range of applications, from traditional catalysis to advanced energy conversion technologies like fuel cells and electrolyzers. These advancements are poised to drive our transition toward a sustainable, green, and technologically advanced society.
